# Citation penalties following sexual versus scientific misconduct allegations

**DOI:** 10.1371/journal.pone.0317736

**Published:** 2025-03-05

**Authors:** Giulia Maimone, Gil Appel, Craig R. M. McKenzie, Ayelet Gneezy

**Affiliations:** 1 Anderson School of Management, UCLA, Los Angeles, CA, United States of America; 2 GW School of Business, George Washington University, Washington, D.C., United States of America; 3 Rady School of Management, UC San Diego, La Jolla, CA, United States of America; 4 Department of Psychology, UC San Diego, La Jolla, CA, United States of America; Iuliu Hațieganu University of Medicine and Pharmacy: Universitatea de Medicina si Farmacie Iuliu Hatieganu, ROMANIA

## Abstract

**Background and aim:**

Citations in academia have long been regarded as a fundamental means of acknowledging the contribution of past work and promoting scientific advancement. The aim of this paper was to investigate the impact that misconduct allegations made against scholars have on the citations of their work, comparing allegations of sexual misconduct (*unrelated* to the research merit) and allegations of scientific misconduct (*directly related* to the research merit).

**Methods:**

We collected citation data from the Web of Science (WoS) in 2021, encompassing 31,941 publications from 172 accused and control scholars across 18 disciplines. We also conducted two studies: one on non-academics (N = 231) and one on academics (N = 240).

**Results:**

The WoS data shows that scholars accused of sexual misconduct incur a significant citation decrease in the three years after the accusations become public, while we do not detect a significant citation decrease for scholars accused of scientific misconduct. The study involving non-academics suggests that individuals are more averse to sexual than to scientific misconduct. Finally, contrary to the WoS data findings, a sample of academics indicates they are more likely to cite scholars accused of sexual misconduct than those accused of scientific misconduct.

**Conclusions:**

In the first three years after accusations became public, scholars accused of sexual misconduct incur a larger citation penalty than scholars accused of scientific misconduct. However, when asked to predict their citing behavior, scholars indicated the reverse pattern, suggesting they might mis-predict their behavior or be reluctant to disclose their preferences.

## 1. Introduction

Powerful social movements such as #MeToo have increased both the public’s awareness of the pervasiveness of sexual misconduct across industries [1] and the demand for accountability [2, 3]. Sexual misconduct is defined as unwelcome behaviors of a sexual nature [4] and commonly occurs in the workplace and other situations involving power imbalance. For example, a supervisor might request sexual favors in exchange for granting tangible job-related benefits or subject an employee to physical or verbal conduct of a sexual nature that is so severe or pervasive as to create an abusive work environment [5]. For the victims, the consequences of sexual misconduct, which may include emotional, physical, and professional outcomes, can be devastating [6].

The #MeToo movement did not spare academia [7, 8]. Despite sexual misconduct offenses continuing to go under-reported [e.g., 9, 10], a 2021 report estimates that about 20% of female and 7% of male undergraduates were targets of sexual misconduct during their college years [11]. In the United States, academic institutions abide by Title IX of the Education Amendments Act of 1972—prohibiting sex-based discrimination under any education program—to regulate processes and sanctions for dealing with transgressors and deterring such behaviors. In addition to such formal regulations, individuals commonly express disapproval or call out deviants in informal ways, such as sharing information on social media [12].

In this article, we consider an additional way that disapproval of academics accused of sexual misconduct might manifest itself. Specifically, we investigate the effect of sexual misconduct allegations on the citation rates of the alleged perpetrators’ work. In theory, the purpose of citations is to promote scientific advancement and acknowledge the contribution of past research [13, 14], and the number of citations is assumed to provide a measure of the quality and impact of scholarly work [15–17]. However, evidence suggests that scientists’ citing decisions are sometimes sensitive to factors unrelated to the research’s relevance, quality, and merit [18]. For example, scientists are more likely to cite more visible articles and scholars [19–21], and they are more likely to cite their friends’ research, in part to help their friends and in part to help themselves, because they are more likely to be cited in return [22, 23]. Whether scholars might also *avoid* citing research published by individuals accused of immoral or controversial behavior is unclear.

We considered the impact that sexual misconduct allegations may have on citations for two reasons. First, following #MeToo going viral in October 2017, such allegations became very prevalent [24]. Second, sexual misconduct is unrelated to the relevance and quality of the accused scholar’s scientific work. Suppose citation decisions were based exclusively on a publication’s relevance and merit. In that case, allegations of sexual misconduct should not influence the citation rates of the accused scholars’ research because their transgression does not implicate the merit of their work. However, scholars might avoid citing the research of a colleague accused of sexual misconduct for various reasons, such as signaling disapproval, punishing the alleged perpetrator, or simply avoiding being associated with them, thereby reducing the accused scholar’s citation rates.

This paper compares the citation rates of scholars accused of sexual misconduct with those of control scholars (see S1 File). A decrease, or a slower increase, in citation rates of the accused scholars’ work after the allegations were made public would constitute a citation penalty. To contextualize the size of any potential citation penalty for scholars accused of sexual misconduct, we also compare these scholars’ citation rates with those of researchers accused of scientific misconduct, which is defined as the violation of the standard codes of scholarly conduct and ethical behavior in the publication of professional scientific research (e.g., data fabrication, falsification, and plagiarism [25–28]). Unlike sexual misconduct, scientific misconduct necessarily implicates the concerned research, invalidating its claims [29] and generating legitimate concerns about the integrity of the entire research portfolio of the accused scholar [e.g., 30]. From this perspective, it is reasonable to expect that any citation penalty experienced by scholars accused of sexual misconduct would be *smaller* than that experienced by scholars accused of scientific misconduct.

This paper aimed to determine whether sexual misconduct allegations against scholars negatively impact the citation rates of their published work, and to compare this potential impact with that incurred by scholars accused of scientific misconduct.

## 2. Materials and methods

We collected Web of Science (WoS) data on the academic citations for all publications (peer-reviewed articles and scholarly books) authored by thirty scholars accused of sexual or scientific misconduct (split evenly), encompassing 18 disciplines (see S1 File for details). All accused scholars included in our sample were active researchers in STEM (Science, Technology, Engineering, and Mathematics) or social sciences, having a minimum of 200 citations overall, and were accused of misconduct in 2017 or earlier. To ensure that our findings are not biased due to differential awareness of the two misconduct types, we only included in our sample scholars whose accusations received similar media attention (i.e., cases for which we could find detailed accounts of the allegations online). To accomplish this, we first searched for online news reports on sexual and scientific misconduct in academia to identify relevant cases for our analyses. To validate the integrity of our selection process, we checked each case against the following sources: the Academic Sexual Misconduct Database, which lists sexual misconduct allegations involving faculty and other university employees [31], Retraction Watch, a blog that reports on retractions of scientific papers [32], and Wikipedia’s List of Scientific Misconduct Incidents [33]. Although this inclusion criterion limits the number of misconduct cases in our dataset, it minimizes the risk that researchers’ awareness of the two misconduct types differed systematically.

Next, we matched each accused scholar with five researchers who were not accused of any misconduct at the time of data collection, totaling 150 controls. We matched five controls to each accused scholar to obtain robust but comparable controls. These controls were matched by searching the faculty directory websites of the same academic department as the accused’s at similarly ranked universities (following the Carnegie Classification of Institutions of Higher Education’s R1, R2, and R3 classification). We were interested in researchers that shared the following qualitative similarities with their respective accused: (a) research discipline, (b) research topics, (c) gender, (d) academic rank, (e) similarly ranked university following the Carnegie Classification, and (f) number of total citations (i.e., pre- and post-accusation) at the time of data collection. Note that we identified the matched controls before we could collect and analyze the citations data, meaning we could not access their citation counts and trajectory over the years. As a result, the citation trajectory of some controls during the pre-accusation period might not be optimally parallel to that of their respective accused scholar. To ensure that this feature of our control scholars is not biasing the results, we ran a series of robustness checks that confirmed that our findings are robust to different controls’ specifications, as well as to excluding controls from the analysis (i.e., comparing only the two groups of accused scholars; see S1 File). After identifying the controls and collecting all data, we discovered that eight control researchers (4 for sexual and 4 for scientific misconduct) had at least one retracted paper. We excluded these scholars from our analyses because they might have been treated as if they were accused of scientific misconduct. Our results remain unchanged when we include all 150 controls (see S1 File). We did not preregister our study because we did not have a directional hypothesis. All data, codes, and materials used for collecting and analyzing the data are available on OSF.

**Fig 1:**
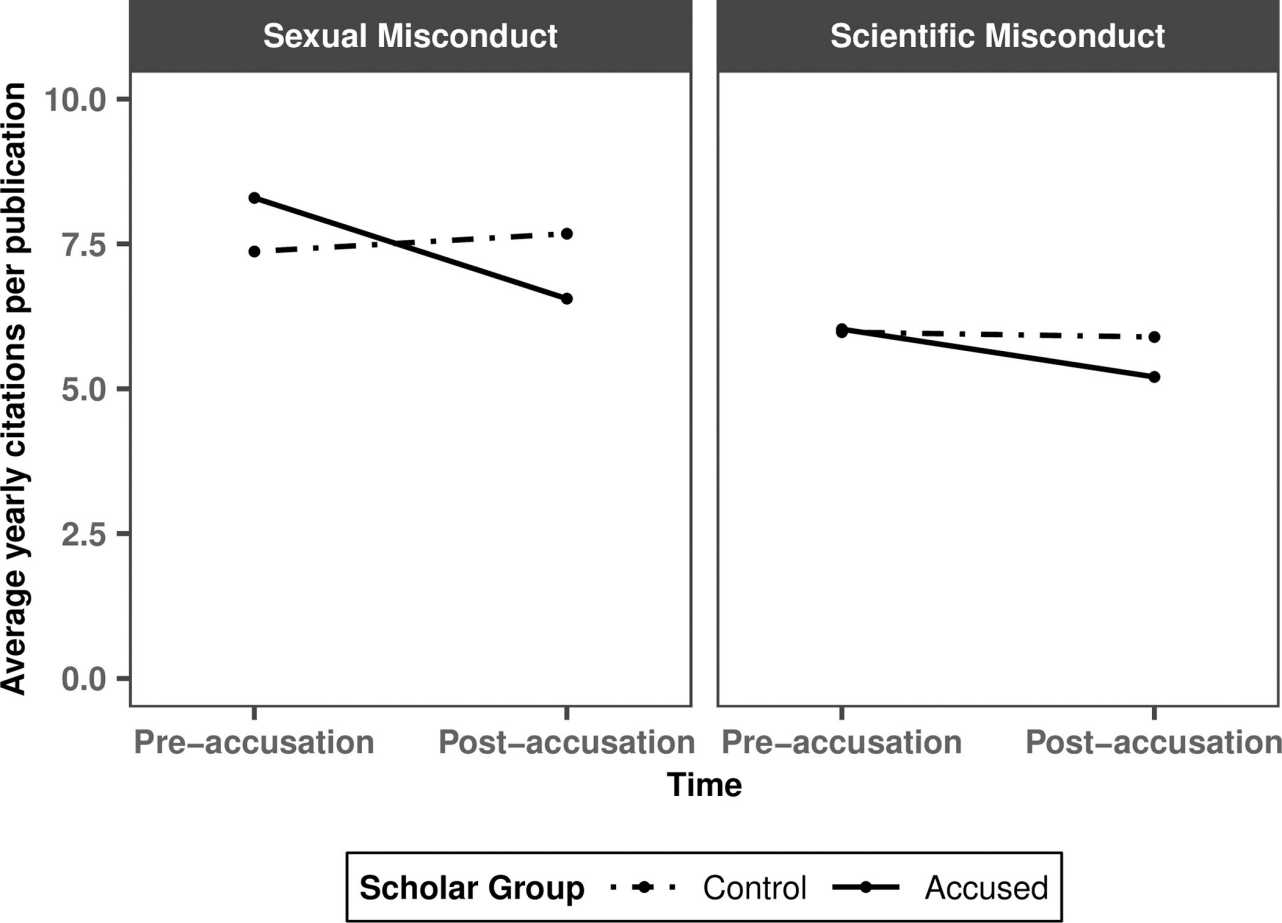
Estimates of the average yearly citations per publication (including all publications) by scholar group, misconduct type, and time.

The primary analyses account for a maximum of 13 years—3 years after the accusations became public and a maximum of 10 years before (later publications might have fewer pre-accusation observations). The results are robust to different specifications of the number of years (5, 8, 12, and 15) in the pre-accusation period (see S1 File). Note that the accusation year was excluded from all analyses and that we completed data collection by the end of June 2021. Across all analyses, we control for the following factors: publication year, total citations per publication (which includes both pre- and post-accusation citations; results are robust when controlling for total citations per publication until the accusation year; see S1 File), number of authors, scholar’s rank, gender, academic discipline, and the year the accusations became public. We also control for the naturally increasing citation trend over time [34, 35]. Hence, in Fig 1, to the extent that a line is flat, citations increased as predicted by the trend, that is, with no penalty or boost. Our results remain robust when we exclude ‘Academic discipline,’ ‘Publication year,’ and ‘Citations trend’ as controls and instead normalize citations by time within each academic discipline (see S1 File). All analyses include standard errors clustered by scholar. Note that in Fig 1 error bars are omitted due to the complexity of visualizing clustered standard errors.

To explore the potential psychological drivers of the citation patterns emerging from the WoS data, we also ran two studies: one on non-academics and one on academics.

In the ‘non-academics’ study, we recruited two hundred fifty-one CloudResearch-approved workers from the US (we did not impose additional restrictions on the sample) through the Amazon MTurk toolkit (https://www.cloudresearch.com). Participants provided informed consent digitally and completed the experiment in exchange for monetary compensation ($0.35 for a 2-minute completion time). Twenty participants were then excluded for failing our attention check, resulting in a final sample of 231. The study was conducted on December 23^rd^, 2021. First, participants were provided with definitions and examples of both scientific and sexual misconduct in academia and were told that both types of misconduct can have different degrees of severity. Next, participants indicated which of the two types of misconduct they thought was (a) more deserving of punishment, (b) more disgusting, and (c) worse than the other. The exact wording of the questions is available in the S1 File (pages 37–39), and the Qualtrics file can be found on OSF. The UCSD Institutional Review Board approved the study (IRB exemption #200195).

For the ‘academics’ study, we recruited one hundred and fifty-six scholars through the Society for Judgment and Decision-Making (https://sjdm.org) mailing list from May 17^th^ to June 7^th^, 2023, and eighty-four scholars through the Advancing Earth and Space Science (https://www.agu.org) community from June 1^st^ to June 21^st^, 2023. All participants (N = 240) provided informed consent digitally and were presented with the definitions and examples of both scientific and sexual misconduct in academia. Next, we asked them to indicate how likely they would be to cite an article authored by a researcher accused of data fabrication (note that the majority of the scholars accused of scientific misconduct in our WoS sample were accused of data fabrication), and how likely they would be to cite an article authored by a researcher accused of sexual harassment (both using a 7-point scale: 1 = I would definitely not cite, 7 = I would definitely cite). We also asked participants to specify which paper—the one authored by a scholar accused of data fabrication or the one authored by a scholar accused of sexual harassment—they would be more likely to cite if forced to choose one, assuming the two were equally relevant. See S1 File and OSF for details. The UCSD Institutional Review Board approved the study (IRB exemption #200195).

## 3. Results

Our final WoS dataset consists of yearly citations for 5,888 publications authored by 30 accused scholars and for 26,053 publications authored by their 142 matched control scholars (N = 31,941), totaling 290,038 observations across a maximum of 13 years.

Our first analysis compares citation rates of the four groups of scholars (i.e., researchers accused of sexual misconduct, researchers accused of scientific misconduct, and their respective controls) before and after the allegations became public through a difference-in-difference-in-differences analysis (Model 1). As expected, this model shows that the citation rates of both control groups are flat (*p*s > .30), indicating no citation penalty. In contrast, citation rates of scholars accused of sexual misconduct decreased both in absolute terms (*b* = -1.74, *t* = -3.86, *p* < .001) and compared to their controls (*b* = -2.05, *t* = -3.65, *p* < .001), indicating a citation penalty (see S1 File for robustness checks). Interestingly, scholars accused of scientific misconduct did not experience a significant citation penalty in absolute terms (*b* = -0.82, *t* = -1.51, *p* = .131) or compared to their controls (*b* = -0.74, *t* = -1.06, *p* = .288). Despite the directionally larger citation penalty observed for scholars accused of sexual vs. scientific misconduct, the three-way interaction of misconduct type (sexual vs. scientific), scholar group (accused vs. control), and time (pre- vs. post-accusation) does not reach significance (*b* = -1.31, *t* = -1.44, *p* = .150).

If scholars are indeed less likely to cite researchers accused of sexual misconduct than those accused of scientific misconduct, one would expect the effect to be stronger for publications where the name of the accused author is more salient. Some publications in our dataset had many authors (the largest had 2,405 authors), and it is reasonable to assume that an author is less identifiable with a publication to the extent that they are one of many. We therefore ran a second model (Model 2), identical to Model 1, with one difference: rather than controlling for the number of authors on a publication, we added this variable as an interaction term. This model reveals a significant three-way interaction of interest (i.e., misconduct type x scholar group x time; *b* = -5.17, *t* = -4.84, *p* < .001), showing that the observed citation penalty is larger for sexual than for scientific misconduct. This model also reveals a significant four-way interaction of misconduct type, scholar group, time, and number of authors (numeric) (*b* = 0.53, *t* = 4.90, *p* < .001), showing that the difference in citation penalty observed for sexual versus scientific misconduct is impacted by the number of authors on a publication. Specifically, the difference in citation penalty between scholars accused of sexual versus scientific misconduct reduces with every additional coauthor.

Given these results, we analyzed the subset of publications with 7 or fewer authors. We chose this cutoff because it is close to the mean number of authors (7.2) in our dataset, and it allows us to keep 75% of the publications for the analysis. We re-ran Model 1 on this new dataset (N = 23,911; the number of scholars did not change). Replicating the findings reported above, this analysis shows that the citation rates of scholars accused of sexual misconduct decreased both in absolute terms (*b* = -1.62, *t* = -3.51, *p* < .001) and compared to their controls (*b* = -2.45, *t* = -3.86, *p* < .001), indicating a citation penalty. Again, this analysis did not detect a citation penalty for scholars accused of scientific misconduct in absolute terms (*b* = -0.35, *t* = -0.69, *p* = .492) or compared to their controls (*b* = 0.46, *t* = 0.83, *p* = .404). Importantly, this model reveals that scholars accused of sexual misconduct incurred a *larger* citation penalty than those accused of scientific misconduct, as suggested by the significant three-way interaction of misconduct type, scholar group, and time (*b* = -2.91, *t* = -3.49, *p* < .001). Consistent with the relatively larger citation penalty for scholars accused of sexual misconduct, we also find a significant head-to-head comparison of citation rates of the two accused groups before and after the allegations became public (*b* = -1.27, *t* = -2.19, *p* = .029), which reveals that the citation rates of scholars accused of sexual misconduct decreased *more* than those of scholars accused of scientific misconduct. These results replicate across a variety of robustness checks (see S1 File). All data, codes, and materials used in the analyses are available on OSF.

**Fig 2:**
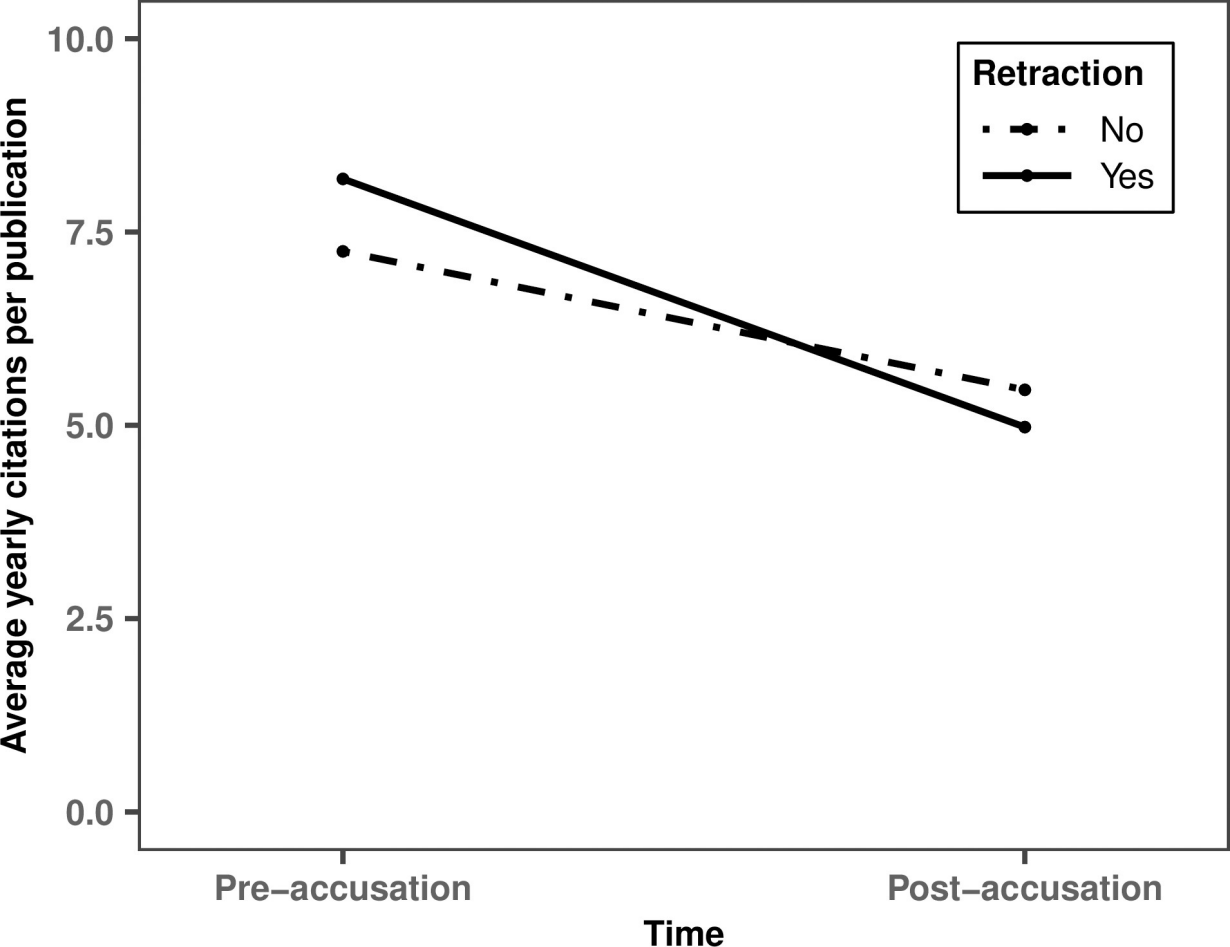
Estimates of average yearly citations of publications by scholars accused of scientific misconduct by retraction status and time.

We consider three unobserved factors that could explain why scholars accused of scientific misconduct incurred a smaller citation penalty. First, these scholars have both retracted and non-retracted publications. Non-retracted publications of scholars accused of scientific misconduct could receive a citation boost if, for example, individuals interpret the absence of retraction as suggesting a publication has been cleared. Alternatively, retracted publications might receive a boost if researchers cite a retracted article when referring to its shortcomings. To test these possibilities, we regressed the average yearly citations per publication of researchers accused of scientific misconduct on retraction status (yes vs. no), time (pre- vs. post-accusation), and their interaction (Model 3). Our analysis reveals that scientific misconduct accusations have no impact on either the citations of retracted publications (*b* = -3.21, *t* = -1.67, *p* = .094) or on the citations of non-retracted publications (*b* = -1.79, *t* = -1.33, *p* = .184). Furthermore, citation rates of retracted and non-retracted publications were not differentially affected (*b* = -1.42, *t* = -0.92, *p* = .357; see Fig 2). Fig 2, like Fig 1, omits error bars due to the complexity of visualizing clustered standard errors.

Second, one could also argue that the two types of misconduct allegations differentially negatively affect the accused’s productivity and, consequently, their citation rates. This explanation seems unlikely because our key dependent variable is the number of yearly citations *per* publication, so any decreased productivity should not directly impact our citation results. Furthermore, we repeated our analyses restricting the dataset to publications published before the accusations became public, which perfectly replicates the results reported in this article (see S1 File), suggesting any potentially reduced productivity is not directly impacting our results. However, reduced productivity might still cause reduced visibility/salience of the scholar in their field, which could negatively affect post-accusation yearly citation rates per publication. Our data only covers three years after the allegations were made public, which seems too short for researchers to forget about their colleagues and their research. Nevertheless, we tested this possibility by repeating our main analysis on citations (Model 1) using the number of yearly publications authored by the scholars in our sample as our dependent variable, serving as a proxy for productivity. This analysis reveals that post-accusation publication rates significantly decreased for scholars accused of sexual misconduct both in absolute terms (*b* = -7.14, *t* = -5.54, *p* < .001) and compared to their controls (*b* = -7.14, *t* = -5.24, *p* < .001). Importantly, publication rates significantly decreased also for scholars accused of scientific misconduct both in absolute terms (*b* = -10.00, *t* = -5.68, *p* < .001) and compared to their controls (*b* = -8.55, *t* = -4.76, *p* < .001). This analysis reveals that publication rates decreased to the same extent for both accused groups, as shown by a non-significant three-way interaction (*b* = 1.41, *t* = 0.62, *p* = .533) and by a non-significant head-to-head comparison (*b* = 2.86, *t* = 1.40, *p* = .161). These results suggest that the post-accusation decline in productivity of the two accused groups cannot explain the difference in their citation penalty, as such decline is similar across groups.

Finally, researchers may be more likely to know about allegations involving sexual versus scientific misconduct if, for example, the former receive more coverage in the news and on social media platforms. Although we cannot completely rule out this possibility, two factors suggest it is unlikely. First, our data included only cases of alleged misconduct that received comparable media attention. Second, while sexual misconduct allegations are often protected by privacy clauses (e.g., non-disclosure agreements), scientific misconduct allegations are not. At the same time, retracted articles are all clearly marked by Google Scholar, Web of Science, and the journal websites.

Why might there be a larger citation penalty for sexual than for scientific misconduct? One possibility is that people find sexual misconduct more reprehensible than scientific misconduct, triggering stronger negative reactions. To test this proposition, we asked a sample of non-academics (N = 231) about their attitudes. We tested the proportion of participants indicating which misconduct type they thought was (a) more deserving of punishment, (b) more disgusting, and (c) worse than the other against 50% with three chi-square tests. These tests revealed that the majority of participants deemed sexual misconduct in academia as more deserving of punishment (76.2%, χ^2^(1) = 63.38, *p* < .001), more disgusting (90.5%, χ^2^(1) = 151.40, *p* < .001), and worse (75.8%, χ^2^(1) = 61.30, *p* < .001) than scientific misconduct. Participants’ gender had no effect on any of these findings (*p*s > .40; see S1 File and OSF for details).

Thus far, we have shown that (a) researchers accused of sexual misconduct incur a larger citation penalty from other researchers, and (b) non-academics consider sexual misconduct as more reprehensible than scientific misconduct. Yet, it is unclear whether researchers believe that scholars accused of sexual misconduct should incur a larger citation penalty than scholars accused of scientific misconduct. They might, for example, feel a need to take (any) action in light of the perceived inadequacy of various policies (e.g., Title IX in the US) and of the justice system to keep sexual offenders accountable and deter future transgressions, or they might want to avoid association with stigmatized behavior. To explore these accounts, we asked academic researchers (N = 240) to self-report their citing behavior. A paired-samples t-test indicated that participants reported they would be more likely to cite the article published by the scholar accused of sexual harassment (M = 4.42, SD = 1.96) than the one published by the scholar accused of data fabrication (M = 2.39, SD = 1.54; *t*(239) = 16.02, *p* < .001). In addition, substantially more participants (85.0%) indicated that, if forced to choose one article to cite, they would opt for the one published by the scholar accused of sexual harassment (χ^2^(1) = 117.60, *p* < .001). (Note that 15.0% of researchers reported being more likely to cite the article published by the scholar accused of data fabrication.) Neither result was affected by participants’ gender (female, male, other) or by field (natural sciences, social sciences, other). See S1 File and OSF for details.

These results are, of course, at odds with the citation data results. If scholars were more likely to cite researchers accused of sexual misconduct over researchers accused of scientific misconduct, then our analyses would have detected a *smaller* citation penalty for researchers accused of sexual misconduct in the citation data. This gap between researchers’ beliefs about their citing behavior and their actual citing behavior suggests two possibilities. One is that researchers are unaware that they are less likely to cite publications authored by scholars accused of sexual misconduct, in which case they may be overestimating their ability to engage in moral decoupling, whereby individuals distinguish a transgressor’s responsibility for an immoral action from their performance on unrelated tasks. The other possibility is that researchers are aware that they are less likely to cite publications authored by scholars accused of sexual misconduct but are unwilling to admit it, perhaps because of self-presentation concerns, as they believe that, normatively, they should not cite those accused of scientific misconduct.

## 4. Discussion

Consistent with the proposition that scholars’ citation decisions are sensitive to factors unrelated to a publication’s scientific merit, all our analyses reveal a significant citation penalty for scholars accused of sexual misconduct. At the same time, we do not detect a significant citation penalty for scholars accused of scientific misconduct. When analyzing the entire dataset, the citation penalty for scholars accused of sexual misconduct was larger than for those accused of scientific misconduct, but the difference did not reach significance. However, when we exclude publications with large numbers of authors, making it more likely to identify the accused author with a publication, the citation penalty incurred by scholars accused of sexual misconduct is significantly larger than that incurred by scholars accused of scientific misconduct. Hence, we conclude that scholars accused of sexual misconduct incur a citation penalty that is *at least as large as* that incurred by scholars accused of scientific misconduct.

These findings are noteworthy because they offer evidence showing that the citation rates of scholars accused of misconduct are negatively affected, even though their alleged misconduct is *unrelated* to the relevance and merit of their research. Several factors could contribute to this finding, such as researchers’ attempt to distance themselves from individuals accused of reprehensible behaviors [36–38]—whether consciously or not—or a desire to punish colleagues for their immoral behavior [12, 39–41]. Importantly, our findings might help raise scholars’ awareness of one way their citation decisions could be biased. This awareness can then guide researchers in making more informed citation choices, which might help eliminate or even reverse the citation penalty gap we observed between scholars accused of sexual and of scientific misconduct.

One limitation of this work is the relatively small number of accused scholars. As mentioned in the Methods section, because we compare the impact of two types of misconduct allegations on citation rates, we attempted to control for numerous confounds. This limited the number of accused scholars who met the criteria for inclusion in our sample. For example, we limited misconduct accusations to cases that were reported by the media and thus were likely to be familiar to scholars in their fields, ensuring that any effect observed in our analyses is not driven by differential awareness of the two misconduct types. Thus, the relatively small number of scholars facilitates comparability across misconduct types. Also, to make sure that we accounted for the limited number of scholars and did not benefit from ‘undue’ power provided by the high number of publications, we clustered the standard errors (SEs) by scholar in all analyses reported in the manuscript and S1 File.

An important question emerging from our research is whether and to what extent our findings generalize. Considering the results of our ‘non-academics’ study, we believe that these results would generalize to other types of misconduct that are unrelated to the accused scholar’s academic work and are considered to be more egregious than scientific misconduct. Also, note that our findings emerge from considering a relatively short period of time (3 years) after the accusations became public. The pattern of results we observed might disappear or even reverse over longer periods of time. It is possible that the citation rates of scholars accused of sexual misconduct decrease more in the short term, as the severity and aversiveness of sexual misconduct might generate intense and impulsive negative emotional reactions that might attenuate with time. On the other hand, a citation penalty triggered by scientific misconduct might be more rationally determined, leading to a slower but constant decrease in citation rates of the accused scholars, which might not be detectable until after 3 years. Whether the penalty gap we observed between sexual and scientific misconduct exists for other misconduct types and whether it would reduce or reverse over extended periods of time remain important open questions for future investigations.

To our knowledge, our paper is the first to systematically compare the ramifications of sexual and scientific misconduct on the citations of alleged perpetrators. Our finding that the citation penalty for sexual misconduct allegations is at least as large as the citation penalty for scientific misconduct adds a new dimension to a body of research showing that citation decisions are sensitive to factors unrelated to a publication’s scientific merit [e.g., 21, 22].

## Supporting information

S1 File (PDF)
