# Supplementary material for: Citation penalties following sexual versus scientific misconduct allegations
Source: PLoS One. 2025 Mar 5;20(3):e0317736. doi: 10.1371/journal.pone.0317736 (PMC11882088; doi:10.1371/journal.pone.0317736)
Supplement: S1 File — (PDF) [file pone.0317736.s001.pdf]

SUPPORTING INFORMATION

Citation Penalties Following Sexual versus Scientific Misconduct Allegations

Data exclusions……p. 2
Accused scholars' summary statistics……p. 2
List of academic disciplines……p. 2
Citations trend by scholar group (raw data)……p. 2
Publications trend by scholar group (raw data)……p. 2
Sample Robustness Checks……p. 3
- Regression tables……p. 4
- Including all 150 controls……p. 8
- Excluding publications published after the accusations became public……p. 9
- Excluding retracted publications……p. 10
- Excluding the only scholar in our sample accused of scientific misconduct who had no retractions—since they were accused of self-plagiarism……p. 11
- Different accusation year for Potti……p. 12
- Including only publications in STEM……p. 13
- LOO-CV analysis on accused scholars……p. 14

Timing Robustness Checks……p. 17
- Regression tables……p. 17
- 5 years before – 3 years after……p. 21
- 8 years before – 3 years after……p. 22
- 12 years before – 3 years after……p. 23
- 15 years before – 3 years after……p. 24

Regression Robustness Checks……p. 25
- Regression tables……p. 25
- Using *total citations up to accusation* as a covariate instead of overall total citations……p. 28
- Normalizing citations data by year within field……p. 29

Control Scholars Robustness Checks……p. 30
- Why we do not use Propensity Score Matching and Synthetic Controls……p. 30
- Regression tables……p. 31
- Excluding all controls from analysis……p. 33
- Merging the two control groups……p. 33
- LOO-CV analysis on control scholars……p. 34

'Non-academics' Study……p. 37
- Material……p. 37
- Results' Graphs……p. 39

'Academics' Study……p. 40
- Material……p. 40
- Results' Graphs……p. 43

DATA EXCLUSIONS

In addition to excluding 8 controls who had at least one retracted paper, we excluded the following publications:
1. Publications coauthored by multiple accused scholars in our sample
2. Publications coauthored by an accused scholar and a control scholar
3. Publications that are retraction reports (i.e., articles that announce a certain publication will be retracted)

ACCUSED SCHOLARS' SUMMARY STATISTICS

ACCUSED SCHOLARS	N	FIELD TYPE	GENDER
**SEXUAL MISCONDUCT**	15	80% Natural Sciences – 20% Social Sciences	100% Males
**SCIENTIFIC MISCONDUCT**	15	60% Natural Sciences – 40% Social Sciences	86.7% Males

LIST OF ACADEMIC DISCIPLINES IN OUR SAMPLE

Astronomy, Astrophysics, Biochemistry, Bioinformatics, Cellular Biology, Chemistry, Economics, Electrical Engineering, Geology, Marketing, Medicine, Microbiology, Molecular Biology, Neuroscience, Political Science, Psychology, Psychology/Neuro, Statistics

CITATIONS TREND BY SCHOLAR GROUP (<u>RAW DATA</u>)

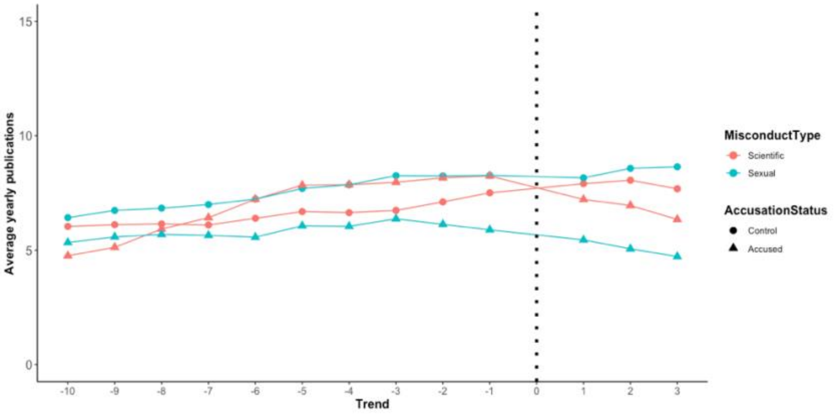

PUBLICATIONS TREND BY SCHOLAR GROUP (<u>RAW DATA</u>)

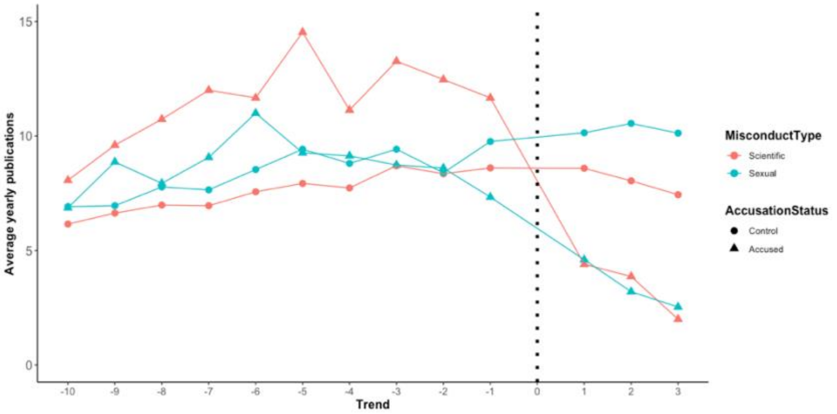

SAMPLE ROBUSTNESS CHECKS

In this section we repeat our analysis on the whole dataset for Model 1 and Model 2, on the few-authors dataset for Model 1, and on the scientific accused only dataset for Model 3 across different sample's specifications. Specifically, we ran each of our models on the following samples:

- Including all 150 controls we initially selected (i.e., 150 controls). Eight of these controls had at least one retracted publication and were removed from the final dataset in the paper.
- Excluding any publications that came out after the year in which the scholars in question were charged with misconduct (i.e., No publ. after accusation).
- Excluding all retracted publications (i.e., No retractions).
- Excluding the only scholar (and their paired controls) accused of scientific misconduct without any retracted publication—as they were accused of self-plagiarism (i.e., No self-plagiarism).
- Using a different accusation year for A. Potti (i.e., A.P. 2010).[1]
- Including only publications in STEM (i.e., Only STEM).

Finally, we ran a cross validation analysis on the accused scholars (leave-one-out cross validation; LOO-CV) and we find that our main results hold when dropping any accused scholar.

[1] In the main analysis we marked Potti's accusation year as 2015, because in that year their accusation was heavily reported in news outlets. However, a first wave of allegations was raised in 2010 even though they did not make it to the media. Our results hold if we consider 2010 as Anil Potti's accusation year (page 12).

Regression Tables

Model 1: 2(Accusation Status: Control vs. Accused) x 2(Misconduct Type: Sexual vs. Scientific) x 2(Timing: Pre- vs. Post-Accusation) – WHOLE DATASET

	Average Yearly Citations per Publication					
	150 controls (N=33,466) (Obs.=304,062)	No publ. after accusations (N=27,825) (Obs.=281,476)	No retractions (N=31,819) (Obs.=289,223)	No self-plagiarism (N=31,345) (Obs.=284,691)	A.P. 2010 (N=31,941) (Obs.=290,038)	Only STEM (N=26,893) (Obs.=243,522)
Intercept	-92.798 (206.118)	-156.811 (179.656)	-206.530 (176.467)	-201.562 (178.016)	-203.909 (176.417)	-182.838 (191.357)
Control	-0.990* (0.391)	-0.963* (0.405)	-0.926* (0.380)	-0.915* (0.379)	-0.926* (0.380)	-0.886* (0.427)
Post-accusation	-1.819*** (0.449)	-1.597*** (0.463)	-1.739*** (0.451)	-1.735*** (0.455)	-1.740*** (0.451)	-1.443** (0.491)
Scientific	-2.554** (0.871)	-2.288** (0.868)	-2.291** (0.862)	-2.189* (0.865)	-2.267** (0.859)	-2.433* (1.161)
Control:Post-accusation	2.123*** (0.547)	2.211*** (0.602)	2.047*** (0.561)	2.033*** (0.561)	2.047*** (0.561)	1.722** (0.608)
Control:Scientific	0.753 (0.578)	0.898 (0.605)	0.912 (0.594)	0.775 (0.601)	0.875 (0.594)	0.670 (0.743)
Post-accusation:Scientific	0.895 (0.609)	0.923 (0.632)	0.942 (0.611)	0.880 (0.632)	0.875 (0.594)	0.948 (0.707)
Control:Post-accusation:Scientific	-1.127 (0.912)	-1.320 (0.960)	-1.333 (0.904)	-1.244 (0.928)	-1.306 (0.906)	-1.135 (1.049)

Note: *p<0.05; **p<0.01; ***p<0.001

#All regressions include controls for the same covariates as the main analysis (i.e., publication year, total citations per publication, number of authors, scholar's discipline, rank, gender, the year the accusations became public, and time trend) and SEs clustered by Scholar. See https://osf.io/ycazs/?view_only=bfb1080d756146ba89d21a7ed3daeacf for full regression code, data, and complete output.

Table 1. Regression table for Model 1 Whole Dataset – Sample robustness checks[2]

[2] The relevant model descriptions and specifications are included in the following pages. (1) 150 controls: page 8; (2) No publ. after accusations: page 9; (3) No retractions: page 10; (4) No self-plagiarism: page 11; (5) A.P. 2010: page 12; (6) Only STEM: page 13.

Model 2: Adding 'AuthorCount' as an interaction term – WHOLE DATASET

	Average Yearly Citations per Publication					
	150 controls (N=33,466) (Obs.=304,062)	No publ. after accusations (N=27,825) (Obs.=281,476)	No retractions (N=31,819) (Obs.=289,223)	No self-plagiarism (N=31,345) (Obs.=284,691)	A.P. 2010 (N=31,941) (Obs.=290,038)	Only STEM (N=26,893) (Obs.=243,522)
Intercept	3.386 (184.907)	-57.358 (171.210)	-80.226 (161.932)	-76.060 (163.865)	-76.731 (162.000)	-45.394 (176.940)
Control	-0.329 (0.467)	-0.194 (0.448)	-0.218 (0.448)	-0.208 (0.452)	-0.219 (0.448)	-0.136 (0.503)
Post-accusation	-1.343 (0.698)	-1.165 (0.710)	-1.242 (0.698)	-1.238 (0.701)	-1.242 (0.698)	-0.944 (0.745)
Scientific	-2.648* (1.137)	-2.552* (1.151)	-2.483* (1.193)	-2.382* (1.179)	-2.540* (1.159)	-2.904 (1.522)
AuthorCount	0.169*** (0.048)	0.185*** (0.051)	0.185*** (0.051)	0.184*** (0.051)	0.185*** (0.051)	0.186*** (0.052)
Control:Post-accusation	2.072** (0.777)	2.237** (0.818)	2.078** (0.783)	2.059** (0.783)	2.077** (0.783)	1.806* (0.833)
Control:Scientific	0.973 (0.991)	1.080 (1.005)	1.021 (1.051)	0.914 (1.041)	1.062 (1.016)	0.884 (1.260)
Post-accusation:Scientific	1.341 (0.841)	1.450 (0.852)	1.447 (0.846)	1.416 (0.870)	1.431 (0.846)	1.838 (0.958)
Control:AuthorCount	-0.101* (0.051)	-0.108 (0.057)	-0.107 (0.056)	-0.107 (0.056)	-0.107 (0.056)	-0.109 (0.058)
Post-accusation:AuthorCount	-0.088 (0.074)	-0.090 (0.075)	-0.089 (0.074)	-0.089 (0.074)	-0.089 (0.074)	-0.088 (0.075)
Scientific:AuthorCount	0.013 (0.108)	0.037 (0.115)	0.019 (0.121)	0.018 (0.116)	0.031 (0.114)	0.037 (0.124)
Control:Post-accusation:Scientific	-4.842*** (1.124)	-5.738*** (1.097)	-5.190*** (1.067)	-5.223*** (1.092)	-5.173*** (1.069)	-5.833*** (1.187)
Control:Post-accusation:AuthorCount	0.050 (0.076)	0.042 (0.077)	0.044 (0.077)	0.045 (0.077)	0.044 (0.077)	0.043 (0.078)
Control:Scientific:AuthorCount	-0.062 (0.112)	-0.066 (0.120)	-0.050 (0.126)	-0.050 (0.121)	-0.062 (0.119)	-0.065 (0.131)
Post-accusation:Scientific:AuthorCount	-0.077 (0.089)	-0.094 (0.090)	-0.088 (0.093)	-0.088 (0.090)	-0.090 (0.090)	-0.127 (0.092)
Control:Post-accusation:Scientific:AuthorCount	0.518*** (0.107)	0.637*** (0.106)	0.530*** (0.111)	0.531*** (0.109)	0.532*** (0.109)	0.572*** (0.111)

Note: *p<0.05; **p<0.01; ***p<0.001

All regressions include controls for the same covariates as the main analysis (i.e., publication year, total citations per publication, scholar's discipline, rank, gender, the year the accusations became public, and time trend) and SEs clustered by Scholar. See https://osf.io/ycazs/?view_only=bfb1080d756146ba89d21a7ed3daeacf for full regression code, data, and complete output.

Table 2. Regression table for Model 2 Whole Dataset – Sample robustness checks[3]

[3] The relevant model descriptions and specifications are included in the following pages. (1) 150 controls: page 8; (2) No publ. after accusations: page 9; (3) No retractions: page 10; (4) No self-plagiarism: page 11; (5) A.P. 2010: page 12; (6) Only STEM: page 13.

Model 1: 2(Accusation Status: Control vs. Accused) x 2(Misconduct Type: Sexual vs. Scientific) x 2(Timing: Pre- vs. Post-Accusation) – FEW AUTHORS DATASET

	Average Yearly Citations per Publication					
	150 controls (N=25,022) (Obs.=240,134)	No publ. after accusations (N=21,372) (Obs.=223,742)	No retractions (N=23,817) (Obs.=228,449)	No self-plagiarism (N=23,315) (Obs.=223,695)	A.P. 2010 (N=23,911) (Obs.=229,042)	Only STEM (N=19,169) (Obs.=184,890)
Intercept	108.952 (182.132)	34.066 (155.908)	24.605 (151.360)	23.898 (153.637)	25.410 (151.267)	72.093 (167.143)
Control	-0.712 (0.431)	-0.610 (0.390)	-0.621 (0.385)	-0.612 (0.388)	-0.621 (0.385)	-0.533 (0.449)
Post-accusation	-1.693*** (0.464)	-1.546** (0.472)	-1.620*** (0.463)	-1.620*** (0.467)	-1.622*** (0.463)	-1.309** (0.507)
Scientific	-2.789*** (0.811)	-2.575*** (0.761)	-2.543*** (0.764)	-2.440** (0.764)	-2.546*** (0.756)	-2.579* (1.030)
Control:Post-accusation	2.487*** (0.620)	2.576*** (0.668)	2.450*** (0.635)	2.440*** (0.635)	2.450*** (0.635)	2.249** (0.711)
Control:Scientific	0.588 (0.623)	0.638 (0.597)	0.637 (0.604)	0.518 (0.603)	0.628 (0.597)	0.263 (0.754)
Post-accusation:Scientific	1.237* (0.582)	1.273* (0.592)	1.294* (0.574)	1.245* (0.603)	1.273* (0.582)	1.549* (0.663)
Control:Post-accusation:Scientific	-2.633** (0.874)	-2.969*** (0.873)	-2.929*** (0.826)	-2.889*** (0.852)	-2.908*** (0.833)	-3.304*** (0.950)

Note: *p<0.05; **p<0.01; ***p<0.001

All regressions include controls for the same covariates as the main analysis (i.e., publication year, total citations per publication, number of authors, scholar's discipline, rank, gender, the year the accusations became public, and time trend) and SEs clustered by Scholar. See https://osf.io/ycazs/?view_only=bfb1080d756146ba89d21a7ed3daeacf for full regression code, data, and complete output.

Table 3. Regression table for Model 1 Few Authors Dataset – Sample robustness checks[4]

[4] The relevant model descriptions and specifications are included in the following pages. (1) 150 controls: page 8; (2) No publ. after accusations: page 9; (3) No retractions: page 10; (4) No self-plagiarism: page 11; (5) A.P. 2010: page 12; (6) Only STEM: page 13.

Model 3: 2(Retraction Status: No vs. Yes) x 2(Timing: Pre- vs. Post-Accusation) – SCIENTIFIC ACCUSED ONLY WHOLE DATASET

	Average Yearly Citations per Publication					
	150 controls (N=3,056) (Obs.=28,228)	No publ. after accusations (N=2,902) (Obs.=27,884)	No retractions	No self-plagiarism (N=2,924) (Obs.=27,418)	A.P. 2010 (N=3,056) (Obs.=28,228)	Only STEM (N=2,491) (Obs.=23,394)
Intercept	-419.693 (434.185)	-481.638 (448.482)		-442.990 (439.374)	-419.693 (434.185)	-100.189 (181.156)
Post-accusation	-3.210 (1.918)	-3.193 (1.915)		-3.223 (1.942)	-3.210 (1.918)	-3.366 (2.969)
NoRetraction	-0.937 (1.395)	-0.921 (1.398)		-0.867 (1.394)	-0.937 (1.395)	-2.015 (1.855)
Post-accusation:NoRetraction	1.419 (1.540)	1.556 (1.523)		1.371 (1.539)	1.419 (1.540)	1.547 (2.635)

Note: *p<0.05; **p<0.01; ***p<0.001

All regressions include controls for the same covariates as the main analysis (i.e., publication year, total citations per publication, number of authors, scholar's discipline, rank, gender, the year the accusations became public, and time trend) and SEs clustered by Scholar. See https://osf.io/ycazs/?view_only=bfb1080d756146ba89d21a7ed3daeacf for full regression code, data, and complete output.

Table 4. Regression table for Model 3 Scientific Accused Only Dataset – Sample robustness checks[5]

[5] The relevant model descriptions and specifications are included in the following pages. (1) 150 controls: page 8; (2) No publ. after accusations: page 9; (3) No retractions: page 10; (4) No self-plagiarism: page 11; (5) A.P. 2010: page 12; (6) Only STEM: page 13.

Including all 150 controls

- *Model 1 WHOLE DATASET* -
We regress average yearly citations per publication on Scholar Group (Control vs. Accused), Misconduct Type (Sexual vs. Scientific), Time (Pre- vs. Post-Accusations), and their interactions, controlling for publication year, total citations per publication, number of authors, scholar's discipline, rank, gender, the year the accusations became public, and time trend. The citation rates of scholars accused of sexual misconduct decreased both in absolute terms (b = -1.82, t = -4.06, p < .001) and compared to their controls (b = -2.12, t = -3.88, p < .001), indicating a citation penalty. However, this analysis indicates that scholars accused of scientific misconduct did not experience a citation penalty in absolute terms (b = -0.92, t = -1.70, p = .089) or compared to their controls (b = -1.00, t = -1.37, p = .170). Despite the qualitatively larger citation penalty for those accused of sexual vs. scientific misconduct, the three-way interaction is not significant (b = -1.13, t = -1.24, p = .217).

- *Model 2 WHOLE DATASET* -
We regress average yearly citations per publication on Scholar Group (Control vs. Accused), Misconduct Type (Sexual vs. Scientific), Time (Pre- vs. Post-Accusations), number of authors (numeric), and their interactions, controlling for publication year, total citations per publication, scholar's discipline, rank, gender, the year the accusations became public, and time trend. This model reveals both a significant three-way interaction of interest (b = -4.84, t = -4.31, p < .001), showing that the citation penalty is larger for sexual vs. scientific misconduct, and a significant four-way interaction (b = 0.52, t = 4.83, p < .001).

- *Model 1 FEW AUTHORS DATASET* -
We regress average yearly citations per publication on Scholar Group (Control vs. Accused), Misconduct Type (Sexual vs. Scientific), Time (Pre- vs. Post-Accusations), and their interactions, controlling for publication year, total citations per publication, number of authors, scholar's discipline, rank, gender, the year the accusations became public, and time trend. The citation rates of scholars accused of sexual misconduct decreased both in absolute terms (b = -1.69, t = -3.65, p < .001) and compared to their controls (b = -2.49, t = -4.01, p < .001), indicating a citation penalty. However, this analysis indicates that scholars accused of scientific misconduct did not experience a citation penalty in absolute terms (b = -0.46, t = -0.90, p = .368) or compared to their controls (b = 0.15, t = 0.23, p = .817). Importantly, this model reveals that scholars accused of sexual misconduct received a *larger* citation penalty than those accused of scientific misconduct, shown by both a significant two-way interaction (b = -1.24, t = -2.13, p = .033) and by a significant three-way interaction (b = -2.63, t = -3.01, p = .003).

- *Model 3 ONLY ACCUSED OF SCIENTIFIC MISCONDUCT DATASET* -
We regress average yearly citations of researchers accused of scientific misconduct on Retraction Status (Yes vs. No), Time (Pre- vs. Post-accusation), and their interaction, controlling for publication year, total citations per publication, number of authors, scholar's discipline, rank, gender, the year the accusations became public, and time trend. The citations of retracted publications did not significantly decrease (b = -3.21, t = -1.67, p = .094), nor did the citations of non-retracted publications (b = -1.79, t = -1.33, p = .184). The citations of the retracted and non-retracted publications decreased to the same extent (b = -1.42, t = -0.92, p = .357).

Excluding publications published after the accusations became public

- *Model 1 WHOLE DATASET* -
We regress average yearly citations per publication on Scholar Group (Control vs. Accused), Misconduct Type (Sexual vs. Scientific), Time (Pre- vs. Post-Accusations), and their interactions, controlling for publication year, total citations per publication, number of authors, scholar's discipline, rank, gender, the year the accusations became public, and time trend. The citation rates of scholars accused of sexual misconduct decreased both in absolute terms (b = -1.60, t = -3.45, p < .001) and compared to their controls (b = -2.21, t = -3.67, p < .001), indicating a citation penalty. However, this analysis indicates that scholars accused of scientific misconduct did not experience a citation penalty in absolute terms (b = -0.67, t = -1.19, p = .234) or compared to their controls (b = -0.89, t = -1.23, p = .220). Despite the qualitatively larger citation penalty for those accused of sexual vs. scientific misconduct, the three-way interaction is not significant (b = -1.32, t = -1.38, p = .169).

- *Model 2 WHOLE DATASET* -
We regress average yearly citations per publication on Scholar Group (Control vs. Accused), Misconduct Type (Sexual vs. Scientific), Time (Pre- vs. Post-Accusations), number of authors (numeric), and their interactions, controlling for publication year, total citations per publication, scholar's discipline, rank, gender, the year the accusations became public, and time trend. This model reveals both a significant three-way interaction of interest (b = -5.74, t = -5.23, p < .001), showing that the citation penalty is larger for sexual vs. scientific misconduct, and a significant four-way interaction (b = 0.64, t = 6.03, p < .001).

- *Model 1 FEW AUTHORS DATASET* -
We regress average yearly citations per publication on Scholar Group (Control vs. Accused), Misconduct Type (Sexual vs. Scientific), Time (Pre- vs. Post-Accusations), and their interactions, controlling for publication year, total citations per publication, number of authors, scholar's discipline, rank, gender, the year the accusations became public, and time trend. The citation rates of scholars accused of sexual misconduct decreased both in absolute terms (b = -1.55, t = -3.28, p = .001) and compared to their controls (b = -2.58, t = -3.86, p < .001), indicating a citation penalty. However, this analysis indicates that scholars accused of scientific misconduct did not experience a citation penalty in absolute terms (b = -0.27, t = -0.53, p = .599) or compared to their controls (b = 0.39, t = 0.70, p = .486). Importantly, this model reveals that scholars accused of sexual misconduct received a *larger* citation penalty than those accused of scientific misconduct, shown by both a significant two-way interaction (b = -1.27, t = -2.15, p = .031) and by a significant three-way interaction (b = -2.97, t = -3.40, p < .001).

- *Model 3 ONLY ACCUSED OF SCIENTIFIC MISCONDUCT DATASET* -
We regress average yearly citations of researchers accused of scientific misconduct on Retraction Status (Yes vs. No), Time (Pre- vs. Post-accusation), and their interaction, controlling for publication year, total citations per publication, number of authors, scholar's discipline, rank, gender, the year the accusations became public, and time trend. The citations of retracted publications did not significantly decrease (b = -3.19, t = -1.67, p = .096), nor did the citations of non-retracted publications (b = -1.64, t = -1.23, p = .219). The citations of the retracted and non-retracted publications decreased to the same extent (b = -1.56, t = -1.02, p = .307).

Excluding retracted publications

- *Model 1 WHOLE DATASET* -
We regress average yearly citations per publication on Scholar Group (Control vs. Accused), Misconduct Type (Sexual vs. Scientific), Time (Pre- vs. Post-Accusations), and their interactions, controlling for publication year, total citations per publication, number of authors, scholar's discipline, rank, gender, the year the accusations became public, and time trend. The citation rates of scholars accused of sexual misconduct decreased both in absolute terms (b = -1.74, t = -3.85, p < .001) and compared to their controls (b = -2.05, t = -3.65, p < .001), indicating a citation penalty. However, this analysis indicates that scholars accused of scientific misconduct did not experience a citation penalty in absolute terms (b = -0.80, t = -1.47, p = .141) or compared to their controls (b = -0.71, t = -1.03, p = .305). Despite the qualitatively larger citation penalty for those accused of sexual vs. scientific misconduct, the three-way interaction is not significant (b = -1.33, t = -1.47, p = .141).

- *Model 2 WHOLE DATASET* -
We regress average yearly citations per publication on Scholar Group (Control vs. Accused), Misconduct Type (Sexual vs. Scientific), Time (Pre- vs. Post-Accusations), number of authors (numeric), and their interactions, controlling for publication year, total citations per publication, scholar's discipline, rank, gender, the year the accusations became public, and time trend. This model reveals both a significant three-way interaction of interest (b = -5.19, t = -4.86, p < .001), showing that the citation penalty is larger for sexual vs. scientific misconduct, and a significant four-way interaction (b = 0.53, t = 4.76, p < .001).

- *Model 1 FEW AUTHORS DATASET* -
We regress average yearly citations per publication on Scholar Group (Control vs. Accused), Misconduct Type (Sexual vs. Scientific), Time (Pre- vs. Post-Accusations), and their interactions, controlling for publication year, total citations per publication, number of authors, scholar's discipline, rank, gender, the year the accusations became public, and time trend. The citation rates of scholars accused of sexual misconduct decreased both in absolute terms (b = -1.62, t = -3.50, p < .001) and compared to their controls (b = -2.45, t = -3.86, p < .001), indicating a citation penalty. However, this analysis indicates that scholars accused of scientific misconduct did not experience a citation penalty in absolute terms (b = -0.33, t = -0.65, p = .515) or compared to their controls (b = 0.48, t = 0.89, p = .376). Importantly, this model reveals that scholars accused of sexual misconduct received a *larger* citation penalty than those accused of scientific misconduct, shown by both a significant two-way interaction (b = -1.29, t = -2.25, p = .024) and by a significant three-way interaction (b = -2.93, t = -3.54, p < .001).

- *Model 3 ONLY ACCUSED OF SCIENTIFIC MISCONDUCT DATASET* -

In this robustness test we exclude all the retracted articles, consequently, we cannot run this analysis as it contrasts retracted vs. not retracted articles.

Excluding the only scholar in our sample accused of scientific misconduct who had no retractions—since they were accused of self-plagiarism

- *Model 1 WHOLE DATASET* -
We regress average yearly citations per publication on Scholar Group (Control vs. Accused), Misconduct Type (Sexual vs. Scientific), Time (Pre- vs. Post-Accusations), and their interactions, controlling for publication year, total citations per publication, number of authors, scholar's discipline, rank, gender, the year the accusations became public, and time trend. The citation rates of scholars accused of sexual misconduct decreased both in absolute terms (b = -1.74, t = -3.81, p < .001) and compared to their controls (b = -2.03, t = -3.63, p < .001), indicating a citation penalty. However, this analysis indicates that scholars accused of scientific misconduct did not experience a citation penalty in absolute terms (b = -0.86, t = -1.52, p = .130) or compared to their controls (b = -0.79, t = -1.08, p = .278). Despite the qualitatively larger citation penalty for those accused of sexual vs. scientific misconduct, the three-way interaction is not significant (b = -1.24, t = -1.34, p = .180).

- *Model 2 WHOLE DATASET* -
We regress average yearly citations per publication on Scholar Group (Control vs. Accused), Misconduct Type (Sexual vs. Scientific), Time (Pre- vs. Post-Accusations), number of authors (numeric), and their interactions, controlling for publication year, total citations per publication, scholar's discipline, rank, gender, the year the accusations became public, and time trend. This model reveals both a significant three-way interaction of interest (b = -5.22, t = -4.78, p < .001), showing that the citation penalty is larger for sexual vs. scientific misconduct, and a significant four-way interaction (b = 0.53, t = 4.87, p < .001).

- *Model 1 FEW AUTHORS DATASET* -
We regress average yearly citations per publication on Scholar Group (Control vs. Accused), Misconduct Type (Sexual vs. Scientific), Time (Pre- vs. Post-Accusations), and their interactions, controlling for publication year, total citations per publication, number of authors, scholar's discipline, rank, gender, the year the accusations became public, and time trend. The citation rates of scholars accused of sexual misconduct decreased both in absolute terms (b = -1.62, t = -3.47, p < .001) and compared to their controls (b = -2.44, t = -3.84, p < .001), indicating a citation penalty. However, this analysis indicates that scholars accused of scientific misconduct did not experience a citation penalty in absolute terms (b = -0.38, t = -0.70, p = .483) or compared to their controls (b = 0.45, t = 0.77, p = .439). Importantly, this model reveals that scholars accused of sexual misconduct received a *larger* citation penalty than those accused of scientific misconduct, shown by both a significant two-way interaction (b = -1.24, t = -2.06, p = .039) and by a significant three-way interaction (b = -2.89, t = -3.39, p < .001).

- *Model 3 ONLY ACCUSED OF SCIENTIFIC MISCONDUCT DATASET* -
We regress average yearly citations of researchers accused of scientific misconduct on Retraction Status (Yes vs. No), Time (Pre- vs. Post-accusation), and their interaction, controlling for publication year, total citations per publication, number of authors, scholar's discipline, rank, gender, the year the accusations became public, and time trend. The citations of retracted publications did not significantly decrease (b = -3.22, t = -1.66, p = .097), nor did the citations of non-retracted publications (b = -1.85, t = -1.34, p = .179). The citations of the retracted and non-retracted publications decreased to the same extent (b = -1.37, t = -0.89, p = .373).

2010 as Accusation Year of A. Potti

- *Model 1 WHOLE DATASET* -
We regress average yearly citations per publication on Scholar Group (Control vs. Accused), Misconduct Type (Sexual vs. Scientific), Time (Pre- vs. Post-Accusations), and their interactions, controlling for publication year, total citations per publication, number of authors, scholar's discipline, rank, gender, the year the accusations became public, and time trend. The citation rates of scholars accused of sexual misconduct decreased both in absolute terms (b = -1.74, t = -3.86, p < .001) and compared to their controls (b = -2.05, t = -3.65, p < .001), indicating a citation penalty. However, this analysis indicates that scholars accused of scientific misconduct did not experience a citation penalty in absolute terms (b = -0.82, t = -1.51, p = .131) or compared to their controls (b = -0.74, t = -1.06, p = .288). Despite the qualitatively larger citation penalty for those accused of sexual vs. scientific misconduct, the three-way interaction is not significant (b = -1.31, t = -1.44, p = .149).

- *Model 2 WHOLE DATASET* -
We regress average yearly citations per publication on Scholar Group (Control vs. Accused), Misconduct Type (Sexual vs. Scientific), Time (Pre- vs. Post-Accusations), number of authors (numeric), and their interactions, controlling for publication year, total citations per publication, scholar's discipline, rank, gender, the year the accusations became public, and time trend. This model reveals both a significant three-way interaction of interest (b = -5.17, t = -4.84, p < .001), showing that the citation penalty is larger for sexual vs. scientific misconduct, and a significant four-way interaction (b = 0.53, t = 4.90, p < .001).

- *Model 1 FEW AUTHORS DATASET* -
We regress average yearly citations per publication on Scholar Group (Control vs. Accused), Misconduct Type (Sexual vs. Scientific), Time (Pre- vs. Post-Accusations), and their interactions, controlling for publication year, total citations per publication, number of authors, scholar's discipline, rank, gender, the year the accusations became public, and time trend. The citation rates of scholars accused of sexual misconduct decreased both in absolute terms (b = -1.62, t = -3.51, p < .001) and compared to their controls (b = -2.45, t = -3.86, p < .001), indicating a citation penalty. However, this analysis indicates that scholars accused of scientific misconduct did not experience a citation penalty in absolute terms (b = -0.35, t = -0.69, p = .492) or compared to their controls (b = 0.46, t = 0.83, p = .404). Importantly, this model reveals that scholars accused of sexual misconduct received a *larger* citation penalty than those accused of scientific misconduct, shown by both a significant two-way interaction (b = -1.27, t = -2.19, p = .029) and by a significant three-way interaction (b = -2.91, t = -3.49, p < .001).

- *Model 3 ONLY ACCUSED OF SCIENTIFIC MISCONDUCT DATASET* -
We regress average yearly citations of researchers accused of scientific misconduct on Retraction Status (Yes vs. No), Time (Pre- vs. Post-accusation), and their interaction, controlling for publication year, total citations per publication, number of authors, scholar's discipline, rank, gender, the year the accusations became public, and time trend. The citations of retracted publications did not significantly decrease (b = -3.21, t = -1.67, p = .094), nor did the citations of non-retracted publications (b = -1.79, t = -1.33, p = .184). The citations of the retracted and non-retracted publications decreased to the same extent (b = -1.42, t = -0.92, p = .357).

Including only publications in STEM

- *Model 1 WHOLE DATASET* -
We regress average yearly citations per publication on Scholar Group (Control vs. Accused), Misconduct Type (Sexual vs. Scientific), Time (Pre- vs. Post-Accusations), and their interactions, controlling for publication year, total citations per publication, number of authors, scholar's discipline, rank, gender, the year the accusations became public, and time trend. The citation rates of scholars accused of sexual misconduct decreased both in absolute terms (b = -1.44, t = -2.94, p = .003) and compared to their controls (b = -1.72, t = -2.83, p = .005), indicating a citation penalty. However, this analysis indicates that scholars accused of scientific misconduct did not experience a citation penalty in absolute terms (b = -0.50, t = -0.77, p = .440) or compared to their controls (b = -0.59, t = -0.70, p = .483). Despite the qualitatively larger citation penalty for those accused of sexual vs. scientific misconduct, the three-way interaction is not significant (b = -1.13, t = -1.08, p = .279).

- *Model 2 WHOLE DATASET* -
We regress average yearly citations per publication on Scholar Group (Control vs. Accused), Misconduct Type (Sexual vs. Scientific), Time (Pre- vs. Post-Accusations), number of authors (numeric), and their interactions, controlling for publication year, total citations per publication, scholar's discipline, rank, gender, the year the accusations became public, and time trend. This model reveals both a significant three-way interaction of interest (b = -5.83, t = -4.91, p < .001), showing that the citation penalty is larger for sexual vs. scientific misconduct, and a significant four-way interaction (b = 0.57, t = 5.17, p < .001).

- *Model 1 FEW AUTHORS DATASET* -
We regress average yearly citations per publication on Scholar Group (Control vs. Accused), Misconduct Type (Sexual vs. Scientific), Time (Pre- vs. Post-Accusations), and their interactions, controlling for publication year, total citations per publication, number of authors, scholar's discipline, rank, gender, the year the accusations became public, and time trend. The citation rates of scholars accused of sexual misconduct decreased both in absolute terms (b = -1.31, t = -2.58, p = .010) and compared to their controls (b = -2.25, t = -3.16, p = .002), indicating a citation penalty. However, this analysis indicates that scholars accused of scientific misconduct did not experience a citation penalty in absolute terms (b = 0.24, t = 0.41, p = .684) or compared to their controls (b = 1.05, t = 1.65, p = .100). Importantly, this model reveals that scholars accused of sexual misconduct received a *larger* citation penalty than those accused of scientific misconduct, shown by both a significant two-way interaction (b = -1.55, t = -2.34, p = .020) and by a significant three-way interaction (b = -3.30, t = -3.48, p < .001).

- *Model 3 ONLY ACCUSED OF SCIENTIFIC MISCONDUCT DATASET* -
We regress average yearly citations of researchers accused of scientific misconduct on Retraction Status (Yes vs. No), Time (Pre- vs. Post-accusation), and their interaction, controlling for publication year, total citations per publication, number of authors, scholar's discipline, rank, gender, the year the accusations became public, and time trend. The citations of retracted publications did not significantly decrease (b = -3.37, t = -1.13, p = .257), nor did the citations of non-retracted publications (b = -1.82, t = -1.10, p = .272). The citations of the retracted and non-retracted publications decreased to the same extent (b = -1.55, t = -0.59, p = .557).

***Leave One Out – Cross Validation (LOO-CV) analysis on accused scholars** - Model 1 WHOLE DATASET*

	Average Yearly Citations per Publication (Reference: Accused, Sexual)					
	Mean Estimate	SD Estimate	Mean SE	Mean t-value	Mean p-value	% Significant
Intercept	-209.70	69.72	182.46	-1.13	0.26	0.00
Control	-0.92	0.10	0.38	-2.39	0.02	93.33
Post-accusation	-1.74	0.14	0.46	-3.78	0.00	100.00
Scientific	-2.24	0.18	0.88	-2,59	0.02	96.67
Control:Post-accusation	2.04	0.20	0.57	3.58	0.00	100.00
Control:Scientific	0.87	0.14	0.60	1.44	0.16	0.00
Post-accusation:Scientific	0.91	0.14	0.62	1.47	0.16	3.33
Control:Post-accusation:Scientific	-1.31	0.25	0.92	-1.42	0.17	0.00

	Average Yearly Citations per Publication (Reference: Accused, Scientific)					
	Mean Estimate	SD Estimate	Mean SE	Mean t-value	Mean p-value	% Significant
Intercept	-211.95	69.61	182.00	-1.15	0.26	0.00
Control	-0.05	0.10	0.46	-0.11	0.87	0.00
Post-accusation	-0.83	0.15	0.55	-1.50	0.15	3.33
Sexual	2.24	0.18	0.88	2.59	0.02	96.67
Control:Post-accusation	0.74	0.15	0.71	1.04	0.30	0.00
Control: Sexual	-0.87	0.14	0.60	-1.44	0.16	0.00
Post-accusation: Sexual	-0.91	0.14	0.62	-1.47	0.16	3.33
Control:Post-accusation: Sexual	1.31	0.25	0.92	1.42	0.17	0.00

	Average Yearly Citations per Publication (Reference: Control, Sexual)					
	Mean Estimate	SD Estimate	Mean SE	Mean t-value	Mean p-value	% Significant
Intercept	-210.62	69.73	182.48	-1.14	0.26	0.00
Accused	0.92	0.10	0.38	2.39	0.02	93.33
Post-accusation	0.31	0.11	0.37	0.84	0.42	3.33
Scientific	-1.38	0.17	0.86	-1.63	0.12	6.67
Accused:Post-accusation	-2.04	0.20	0.57	-3.58	0.00	100.00
Accused:Scientific	-0.87	0.14	0.60	-1.44	0.16	0.00
Post-accusation:Scientific	-0.40	0.23	0.69	-0.58	0.56	0.00
Accused:Post-accusation:Scientific	1.31	0.25	0.92	1.42	0.17	0.00

	Average Yearly Citations per Publication (Reference: Control, Scientific)					
	Mean Estimate	SD Estimate	Mean SE	Mean t-value	Mean p-value	% Significant
Intercept	-211.99	69.63	181.99	-1.15	0.26	0.00
Accused	0.05	0.10	0.46	0.11	0.87	0.00
Post-accusation	-0.09	0.12	0.53	-0.18	0.82	0.00
Sexual	1.38	0.17	0.86	1.63	0.12	6.67
Accused:Post-accusation	2.04	0.20	0.57	3.58	0.00	100.00
Accused: Sexual	0.87	0.14	0.60	1.44	0.16	0.00
Post-accusation: Sexual	0.40	0.23	0.69	0.58	0.56	0.00
Accused:Post-accusation: Sexual	-1.31	0.25	0.92	-1.42	0.17	0.00

Leave One Out – Cross Validation (LOO-CV) analysis on accused scholars - *Model 2 WHOLE DATASET*

	Average Yearly Citations per Publication (Reference: Accused, Sexual)					
	Mean Estimate	SD Estimate	Mean SE	Mean t-value	Mean p-value	% Significant
Intercept	-82.47	40.73	168.00	-0.48	0.64	0.00
Control	-0.22	0.12	0.45	-0.47	0.60	0.00
Post-accusation	-1.24	0.23	0.70	-1.77	0.09	6.67
Scientific	-2.52	0.22	1.18	-2.15	0.03	90.00
AuthorCount	0.18	0.02	0.05	3.71	0.00	100.00
Control:Post-accusation	2.08	0.26	0.78	2.64	0.01	96.67
Control:Scientific	1.06	0.27	1.03	1.02	0.32	0.00
Post-accusation:Scientific	1.43	0.20	0.85	1.68	0.10	3.33
Control:AuthorCount	-0.11	0.01	0.06	-1.91	0.07	3.33
Post-accusation:AuthorCount	-0.09	0.02	0.07	-1.23	0.25	3.33
Scientific:AuthorCount	0.03	0.03	0.11	0.26	0.75	0.00
Control:Post-accusation:Scientific	-1.31	0.25	0.92	-1.42	0.17	0.00
Control:Post-accusation:AuthorCount	0.04	0.02	0.08	0.56	0.55	3.33
Control:Scientific:AuthorCount	-0.06	0.04	0.12	-0.51	0.58	0.00
Post-accusation:Scientific:AuthorCount	-0.09	0.02	0.09	-1.03	0.33	3.33
Control:Post-accusation:Scientific:AuthorCount	0.53	0.04	0.11	4.92	0.00	100.00

Leave One Out – Cross Validation (LOO-CV) analysis on accused scholars - *Model 1 FEW AUTHORS DATASET*

	Average Yearly Citations per Publication (Reference: Accused, Sexual)					
	Mean Estimate	SD Estimate	Mean SE	Mean t-value	Mean p-value	% Significant
Intercept	13.75	68.18	157.12	0.12	0.81	0.00
Control	-0.62	0.11	0.39	-1.59	0.12	3.33
Post-accusation	-1.62	0.15	0.47	-3.45	0.00	100.00
Scientific	-2.51	0.25	0.77	-3.29	0.00	96.67
Control:Post-accusation	2.04	0.20	0.57	3.58	0.00	100.00
Control:Scientific	2.45	0.23	0.64	3.81	0.00	100.00
Post-accusation:Scientific	1.27	0.12	0.59	2.15	0.04	90.00
Control:Post-accusation:Scientific	-2.90	0.27	0.85	-3.43	0.00	100.00

	Average Yearly Citations per Publication (Reference: Accused, Scientific)					
	Mean Estimate	SD Estimate	Mean SE	Mean t-value	Mean p-value	% Significant
Intercept	11.95	68.00	156.69	0.10	0.82	0.00
Control	0.01	0.13	0.49	0.00	0.89	0.00
Post-accusation	-0.35	0.14	0.52	-0.68	0.51	0.00
Sexual	2.51	0.25	0.77	3.29	0.00	96.67
Control:Post-accusation	-0.46	0.15	0.56	-0.82	0.42	0.00
Control: Sexual	-0.63	0.16	0.60	-1.03	0.31	0.00
Post-accusation: Sexual	-1.27	0.12	0.59	-2.15	0.04	90.00
Control:Post-accusation: Sexual	2.90	0.27	0.85	3.43	0.00	100.00

	Average Yearly Citations per Publication (Reference: Control, Sexual)					
	Mean Estimate	SD Estimate	Mean SE	Mean t-value	Mean p-value	% Significant
Intercept	13.13	68.17	157.12	0.11	0.81	0.00
Accused	0.62	0.11	0.39	1.59	0.12	3.33
Post-accusation	0.83	0.12	0.36	2.29	0.03	90.00
Scientific	-1.89	0.21	0.73	-2.62	0.02	93.33
Accused:Post-accusation	-2.45	0.23	0.64	-3.81	0.00	100.00
Accused:Scientific	-0.63	0.16	0.60	-1.03	0.31	0.00
Post-accusation:Scientific	-1.63	0.21	0.61	-2.69	0.01	100.00
Accused:Post-accusation:Scientific	2.90	0.27	0.85	3.43	0.00	100.00

	Average Yearly Citations per Publication (Reference: Control, Scientific)					
	Mean Estimate	SD Estimate	Mean SE	Mean t-value	Mean p-value	% Significant
Intercept	11.24	68.06	156.69	0.10	0.82	0.00
Accused	-0.01	0.13	0.49	0.00	0.89	0.00
Post-accusation	-0.81	0.11	0.34	-2.37	0.02	86.67
Sexual	1.89	0.21	0.73	2.62	0.02	93.33
Accused:Post-accusation	0.46	0.15	0.56	0.82	0.42	0.00
Accused: Sexual	0.63	0.16	0.60	1.03	0.31	0.00
Post-accusation: Sexual	1.63	0.21	0.61	2.69	0.01	100.00
Accused:Post-accusation: Sexual	-2.90	0.27	0.85	-3.43	0.00	100.00

TIMING ROBUSTNESS CHECKS

In this section we test our findings from Model 1, Model 2, and Model 3 including different numbers of years in the 'Pre-accusation' period.
Specifically, we ran each of our models on four different time specifications:

- Including 5 years before the year the accusations became public, and 3 years after (i.e., 5 before – 3 after)
- Including 8 years before the year the accusations became public, and 3 years after (i.e., 8 before – 3 after)
- Including 12 years before the year the accusations became public, and 3 years after (i.e., 12 before – 3 after)
- Including 15 years before the year the accusations became public, and 3 years after (i.e., 15 before – 3 after)

Regression Tables

Model 1: 2(Accusation Status: Control vs. Accused) x 2(Misconduct Type: Sexual vs. Scientific) x 2(Timing: Pre- vs. Post-Accusation) – WHOLE DATASET

	Average Yearly Citations per Publication			
	5 before - 3 after<br>(N=31,941)<br>(Obs.=210,087)	8 before - 3 after<br>(N=31,941)<br>(Obs.=262,072)	12 before - 3 after<br>(N=31,941)<br>(Obs.=313,384)	15 before - 3 after<br>(N=31,941)<br>(Obs.=341,282)
Intercept	-422.996*<br>(203.689)	-271.425<br>(192.022)	-178.548<br>(157.059)	-184.303<br>(138.974)
Control	-0.711<br>(0.445)	-0.861*<br>(0.399)	-0.992**<br>(0.381)	-0.991*<br>(0.393)
Post-accusation	-1.426***<br>(0.332)	-1.755***<br>(0.419)	-1.730***<br>(0.464)	-1.588***<br>(0.466)
Scientific	-1.283<br>(0.820)	-1.889*<br>(0.845)	-2.403**<br>(0.828)	-2.421**<br>(0.770)
Control:Post-accusation	1.738***<br>(0.435)	1.957***<br>(0.526)	2.135***<br>(0.584)	2.161***<br>(0.605)
Control:Scientific	0.082<br>(0.571)	0.575<br>(0.573)	1.034<br>(0.576)	1.146*<br>(0.538)
Post-accusation:Scientific	0.279<br>(0.651)	0.680<br>(0.586)	0.999<br>(0.622)	1.003<br>(0.606)
Control:Post-accusation:Scientific	-0.435<br>(0.854)	-0.991<br>(0.859)	-1.484<br>(0.940)	-1.627<br>(0.962)

Note: *p<0.05; **p<0.01; ***p<0.001

#All regressions include controls for the same covariates as the main analysis (i.e., publication year, total citations per publication, number of authors, scholar's discipline, rank, gender, the year the accusations became public, and time trend) and SEs clustered by Scholar. See https://osf.io/ycazs/?view_only=bfb1080d756146ba89d21a7ed3daeacf for full regression code, data, and complete output.

Table 5. Regression table for Model 1 Whole Dataset – Timing robustness checks[6]

[6] The relevant model descriptions and specifications are included in the following pages. (1) 5 before – 3 after: page 21, (2) 8 before – 3 after: page 22; (3) 12 before – 3 after: page 23; (4) 15 before – 3 after: page 24.

Model 2: Adding 'AuthorCount' as an interaction term – WHOLE DATASET

	Average Yearly Citations per Publication			
	5 before - 3 after (N=31,941) (Obs.=210,087)	8 before - 3 after (N=31,941) (Obs.=262,072)	12 before - 3 after (N=31,941) (Obs.=313,384)	15 before - 3 after (N=31,941) (Obs.=341,282)
Intercept	-286.223 (187.934)	-144.741 (178.354)	-49.443 (141.526)	-53.334 (122.5496)
Control	0.210 (0.378)	0.017 (0.470)	-0.256 (0.469)	-0.237 (0.489)
Post-accusation	-0.716 (0.603)	-1.108 (0.758)	-1.245 (0.706)	-1.103 (0.688)
Scientific	-1.950 (1.137)	-2.281 (1.214)	-2.620* (1.144)	-2.638* (1.122)
AuthorCount	0.178*** (0.043)	0.196** (0.063)	0.197*** (0.054)	0.213*** (0.058)
Control:Post-accusation	1.297* (0.655)	1.720* (0.823)	2.227** (0.798)	2.355** (0.796)
Control:Scientific	1.014 (1.022)	1.052 (1.078)	1.038 (1.016)	1.041 (1.017)
Post-accusation:Scientific	1.247 (0.852)	1.335 (0.940)	1.440 (0.834)	1.434 (0.802)
Control:AuthorCount	-0.127** (0.043)	-0.131* (0.065)	-0.115 (0.060)	-0.122 (0.065)
Post-accusation:AuthorCount	-0.116 (0.072)	-0.113 (0.091)	-0.089 (0.076)	-0.090 (0.076)
Scientific:AuthorCount	0.098 (0.139)	0.053 (0.132)	0.022 (0.119)	0.022 (0.130)
Control:Post-accusation:Scientific	-4.736*** (0.988)	-5.024*** (1.104)	-5.280*** (1.084)	-5.474*** (1.090)
Control:Post-accusation:AuthorCount	0.093 (0.073)	0.078 (0.092)	0.041 (0.078)	0.035 (0.080)
Control:Scientific:AuthorCount	-0.194 (0.141)	-0.115 (0.134)	-0.029 (0.125)	-0.008 (0.137)
Post-accusation:Scientific:AuthorCount	-0.167 (0.129)	-0.114 (0.114)	-0.078 (0.091)	-0.075 (0.093)
Control:Post-accusation:Scientific:AuthorCount	0.630*** (0.136)	0.571*** (0.123)	0.512*** (0.111)	0.507*** (0.114)

Note: *p<0.05; **p<0.01; ***p<0.001

All regressions include controls for the same covariates as the main analysis (i.e., publication year, total citations per publication, scholar's discipline, rank, gender, the year the accusations became public, and time trend) and SEs clustered by Scholar. See https://osf.io/ycazs/?view_only=bfb1080d756146ba89d21a7ed3daeacf for full regression code, data, and complete output.

Table 6. Regression table for Model 2 Whole Dataset – Timing robustness checks[7]

[7] The relevant model descriptions and specifications are included in the following pages. (1) 5 before – 3 after: page 21, (2) 8 before – 3 after: page 22; (3) 12 before – 3 after: page 23; (4) 15 before – 3 after: page 24.

Model 1: 2(Accusation Status: Control vs. Accused) x 2(Misconduct Type: Sexual vs. Scientific) x 2(Timing: Pre- vs. Post-Accusation) – FEW AUTHORS DATASET

	Average Yearly Citations per Publication			
	5 before - 3 after (N=23,911) (Obs.=162,359)	8 before - 3 after (N=23,911) (Obs.=205,318)	12 before - 3 after (N=23,911) (Obs.=249,150)	15 before - 3 after (N=23,911) (Obs.=273,655)
Intercept	-103.956 (191.616)	-13.952 (172.624)	42.339 (128.405)	26.629 (103.678)
Control	-0.213 (0.459)	-0.476 (0.399)	-0.712 (0.384)	-0.688 (0.380)
Post-accusation	-1.366*** (0.348)	-1.629*** (0.431)	-1.594** (0.489)	-1.429** (0.492)
Scientific	-1.849* (0.813)	-2.278** (0.779)	-2.679*** (0.718)	-2.627*** (0.655)
Control:Post-accusation	1.857*** (0.470)	2.240*** (0.588)	2.595*** (0.669)	2.651*** (0.694)
Control:Scientific	-0.123 (0.614)	0.354 (0.602)	0.779 (0.574)	0.847 (0.520)
Post-accusation:Scientific	0.793 (0.452)	1.087* (0.538)	1.337* (0.590)	1.297* (0.569)
Control:Post-accusation:Scientific	-1.885** (0.631)	-2.542*** (0.766)	-3.125*** (0.875)	-3.289*** (0.905)

Note: *p<0.05; **p<0.01; ***p<0.001

#All regressions include controls for the same covariates as the main analysis (i.e., publication year, total citations per publication, number of authors, scholar's discipline, rank, gender, the year the accusations became public, and time trend) and SEs clustered by Scholar. See https://osf.io/ycazs/?view_only=bfb1080d756146ba89d21a7ed3daeacf for full regression code, data, and complete output.

Table 7. Regression table for Model 1 Few Authors Dataset – Timing robustness checks[8]

[8] The relevant model descriptions and specifications are included in the following pages. (1) 5 before – 3 after: page 21, (2) 8 before – 3 after: page 22; (3) 12 before – 3 after: page 23; (4) 15 before – 3 after: page 24.

Model 3: 2(Retraction Status: No vs. Yes) x 2(Timing: Pre- vs. Post-Accusation) – SCIENTIFIC ACCUSED ONLY WHOLE DATASET

	Average Yearly Citations per Publication			
	5 before - 3 after (N=3,056) (Obs.=20,944)	8 before - 3 after (N=3,056) (Obs.=25,808)	12 before - 3 after (N=3,056) (Obs.=30,147)	15 before - 3 after (N=3,056) (Obs.=32,249)
Intercept	-435.121<br>(495.565)	-421.259<br>(445.289)	-390.526<br>(409.672)	-340.127<br>(381.376)
Post-accusation	-1.689<br>(0.949)	-3.209<br>(1.913)	-2.847<br>(1.792)	-2.416<br>(1.625)
NoRetraction	-0.363<br>(1.107)	-1.079<br>(1.426)	-0.899<br>(1.390)	-0.950<br>(1.377)
Post-accusation:NoRetraction	0.705<br>(1.051)	1.513<br>(1.640)	1.408<br>(1.526)	1.452<br>(1.511)

Note: *p<0.05; **p<0.01; ***p<0.001

#All regressions include controls for the same covariates as the main analysis (i.e., publication year, total citations per publication, number of authors, scholar’s discipline, rank, gender, the year the accusations became public, and time trend) and SEs clustered by Scholar. See https://osf.io/ycazs/?view_only=bfb1080d756146ba89d21a7ed3daeacf for full regression code, data, and complete output.

Table 8. Regression table for Model 3 Scientific Accused Only Dataset – Timing robustness checks[9]

[9] The relevant model descriptions and specifications are included in the following pages. (1) 5 before – 3 after: page 21, (2) 8 before – 3 after: page 22; (3) 12 before – 3 after: page 23; (4) 15 before – 3 after: page 24.

5 years before – 3 years after

- *Model 1 WHOLE DATASET* -
We regress average yearly citations per publication on Scholar Group (Control vs. Accused), Misconduct Type (Sexual vs. Scientific), Time (Pre- vs. Post-Accusations), and their interactions, controlling for publication year, total citations per publication, number of authors, scholar's discipline, rank, gender, the year the accusations became public, and time trend. The citation rates of scholars accused of sexual misconduct decreased both in absolute terms (b = -1.43, t = -4.30, p < .001) and compared to their controls (b = -1.74, t = -3.99, p < .001), indicating a citation penalty. However, this analysis indicates that scholars accused of scientific misconduct did not experience a citation penalty in absolute terms (b = -1.15, t = -1.87, p = .061) or compared to their controls (b = -1.30, t = -1.81, p = .070). Despite the qualitatively larger citation penalty for those accused of sexual vs. scientific misconduct, the three-way interaction is not significant (b = -0.44, t = -0.51, p = .611).

- *Model 2 WHOLE DATASET* -
We regress average yearly citations per publication on Scholar Group (Control vs. Accused), Misconduct Type (Sexual vs. Scientific), Time (Pre- vs. Post-Accusations), number of authors (numeric), and their interactions, controlling for publication year, total citations per publication, scholar's discipline, rank, gender, the year the accusations became public, and time trend. This model reveals both a significant three-way interaction of interest (b = -4.74, t = -4.79, p < .001), showing that the citation penalty is larger for sexual vs. scientific misconduct, and a significant four-way interaction (b = 0.63, t = 4.64, p < .001).

- *Model 1 FEW AUTHORS DATASET* -
We regress average yearly citations per publication on Scholar Group (Control vs. Accused), Misconduct Type (Sexual vs. Scientific), Time (Pre- vs. Post-Accusations), and their interactions, controlling for publication year, total citations per publication, number of authors, scholar's discipline, rank, gender, the year the accusations became public, and time trend. The citation rates of scholars accused of sexual misconduct decreased both in absolute terms (b = -1.37, t = -3.93, p < .001) and compared to their controls (b = -1.86, t = -3.95, p < .001), indicating a citation penalty. However, this analysis indicates that scholars accused of scientific misconduct did not experience a citation penalty in absolute terms (b = -0.57, t = -1.42, p = .155) or compared to their controls (b = 0.03, t = 0.07, p = .946). Importantly, this model reveals that scholars accused of sexual misconduct received a *larger* citation penalty than those accused of scientific misconduct, weakly shown by an only marginally significant two-way interaction (b = -0.79, t = -1.76, p = .079) and by a significant three-way interaction (b = -1.89, t = -2.99, p = .003).

- *Model 3 ONLY ACCUSED OF SCIENTIFIC MISCONDUCT DATASET* -
We regress average yearly citations of researchers accused of scientific misconduct on Retraction Status (Yes vs. No), Time (Pre- vs. Post-accusation), and their interaction, controlling for publication year, total citations per publication, number of authors, scholar's discipline, rank, gender, the year the accusations became public, and time trend. The citations of retracted publications did not significantly decrease (b = -1.69, t = -1.78, p = .075), nor did the citations of non-retracted publications (b = -0.98, t = -1.38, p = .168). The citations of the retracted and non-retracted publications decreased to the same extent (b = -0.71, t = -0.67, p = .502).

8 years before – 3 years after

- *Model 1 WHOLE DATASET* -
We regress average yearly citations per publication on Scholar Group (Control vs. Accused), Misconduct Type (Sexual vs. Scientific), Time (Pre- vs. Post-Accusations), and their interactions, controlling for publication year, total citations per publication, number of authors, scholar's discipline, rank, gender, the year the accusations became public, and time trend. The citation rates of scholars accused of sexual misconduct decreased both in absolute terms (b = -1.75, t = -4.18, p < .001) and compared to their controls (b = -1.96, t = -3.72, p < .001), indicating a citation penalty. However, this analysis indicates that while scholars accused of scientific misconduct experienced a citation penalty in absolute terms (b = -1.07, t = -1.98, p = .048), they did not receive a citation penalty compared to their controls (b = -0.97, t = -1.46, p = .144). Despite the qualitatively larger citation penalty for those accused of sexual vs. scientific misconduct, the three-way interaction is not significant (b = -0.99, t = -1.15, p = .249).

- *Model 2 WHOLE DATASET* -
We regress average yearly citations per publication on Scholar Group (Control vs. Accused), Misconduct Type (Sexual vs. Scientific), Time (Pre- vs. Post-Accusations), number of authors (numeric), and their interactions, controlling for publication year, total citations per publication, scholar's discipline, rank, gender, the year the accusations became public, and time trend. This model reveals both a significant three-way interaction of interest (b = -5.02, t = -4.55, p < .001), showing that the citation penalty is larger for sexual vs. scientific misconduct, and a significant four-way interaction (b = 0.57, t = 4.63, p < .001).

- *Model 1 FEW AUTHORS DATASET* -
We regress average yearly citations per publication on Scholar Group (Control vs. Accused), Misconduct Type (Sexual vs. Scientific), Time (Pre- vs. Post-Accusations), and their interactions, controlling for publication year, total citations per publication, number of authors, scholar's discipline, rank, gender, the year the accusations became public, and time trend. The citation rates of scholars accused of sexual misconduct decreased both in absolute terms (b = -1.63, t = -3.78, p < .001) and compared to their controls (b = -2.24, t = -3.81, p < .001), indicating a citation penalty. However, this analysis indicates that scholars accused of scientific misconduct did not experience a citation penalty in absolute terms (b = -0.54, t = -1.17, p = .242) or compared to their controls (b = 0.30, t = 0.60, p = .546). Importantly, this model reveals that scholars accused of sexual misconduct received a *larger* citation penalty than those accused of scientific misconduct, shown by both a significant two-way interaction (b = -1.09, t = -2.02, p = .043) and by a significant three-way interaction (b = -2.54, t = -3.32, p < .001).

- *Model 3 ONLY ACCUSED OF SCIENTIFIC MISCONDUCT DATASET* -
We regress average yearly citations of researchers accused of scientific misconduct on Retraction Status (Yes vs. No), Time (Pre- vs. Post-accusation), and their interaction, controlling for publication year, total citations per publication, number of authors, scholar's discipline, rank, gender, the year the accusations became public, and time trend. The citations of retracted publications did not significantly decrease (b = -3.21, t = -1.68, p = .094), nor did the citations of non-retracted publications (b = -1.70, t = -1.26, p = .208). The citations of the retracted and non-retracted publications decreased to the same extent (b = -1.51, t = -0.92, p = .356).

12 years before – 3 years after

- *Model 1 WHOLE DATASET* -
We regress average yearly citations per publication on Scholar Group (Control vs. Accused), Misconduct Type (Sexual vs. Scientific), Time (Pre- vs. Post-Accusations), and their interactions, controlling for publication year, total citations per publication, number of authors, scholar's discipline, rank, gender, the year the accusations became public, and time trend. The citation rates of scholars accused of sexual misconduct decreased both in absolute terms (b = -1.73, t = -3.73, p < .001) and compared to their controls (b = -2.14, t = -3.66, p < .001), indicating a citation penalty. However, this analysis indicates that scholars accused of scientific misconduct did not experience a citation penalty in absolute terms (b = -0.73, t = -1.28, p = .199) or compared to their controls (b = -0.65, t = -0.90, p = .367). Despite the qualitatively larger citation penalty for those accused of sexual vs. scientific misconduct, the three-way interaction is not significant (b = -1.48, t = -1.58, p = .114).

- *Model 2 WHOLE DATASET* -
We regress average yearly citations per publication on Scholar Group (Control vs. Accused), Misconduct Type (Sexual vs. Scientific), Time (Pre- vs. Post-Accusations), number of authors (numeric), and their interactions, controlling for publication year, total citations per publication, scholar's discipline, rank, gender, the year the accusations became public, and time trend. This model reveals both a significant three-way interaction of interest (b = -5.28, t = -4.87, p < .001), showing that the citation penalty is larger for sexual vs. scientific misconduct, and a significant four-way interaction (b = 0.51, t = 4.62, p < .001).

- *Model 1 FEW AUTHORS DATASET* -
We regress average yearly citations per publication on Scholar Group (Control vs. Accused), Misconduct Type (Sexual vs. Scientific), Time (Pre- vs. Post-Accusations), and their interactions, controlling for publication year, total citations per publication, number of authors, scholar's discipline, rank, gender, the year the accusations became public, and time trend. The citation rates of scholars accused of sexual misconduct decreased both in absolute terms (b = -1.59, t = -3.26, p = .001) and compared to their controls (b = -2.60, t = -3.88, p < .001), indicating a citation penalty. However, this analysis indicates that scholars accused of scientific misconduct did not experience a citation penalty in absolute terms (b = -0.26, t = -0.47, p = .639) or compared to their controls (b = 0.53, t = 0.93, p = .354). Importantly, this model reveals that scholars accused of sexual misconduct received a *larger* citation penalty than those accused of scientific misconduct, shown by both a significant two-way interaction (b = -1.34, t = -2.27, p = .023) and by a significant three-way interaction (b = -3.13, t = -3.57, p < .001).

- *Model 3 ONLY ACCUSED OF SCIENTIFIC MISCONDUCT DATASET* -
We regress average yearly citations of researchers accused of scientific misconduct on Retraction Status (Yes vs. No), Time (Pre- vs. Post-accusation), and their interaction, controlling for publication year, total citations per publication, number of authors, scholar's discipline, rank, gender, the year the accusations became public, and time trend. The citations of retracted publications did not significantly decrease (b = -2.85, t = -1.59, p = .112), nor did the citations of non-retracted publications (b = -1.44, t = -1.31, p = .189). The citations of the retracted and non-retracted publications decreased to the same extent (b = -1.41, t = -0.92, p = .356).

15 years before – 3 years after

- *Model 1 WHOLE DATASET* -
We regress average yearly citations per publication on Scholar Group (Control vs. Accused), Misconduct Type (Sexual vs. Scientific), Time (Pre- vs. Post-Accusations), and their interactions, controlling for publication year, total citations per publication, number of authors, scholar's discipline, rank, gender, the year the accusations became public, and time trend. The citation rates of scholars accused of sexual misconduct decreased both in absolute terms (b = -1.59, t = -3.41, p < .001) and compared to their controls (b = -2.16, t = -3.57, p < .001), indicating a citation penalty. However, this analysis indicates that scholars accused of scientific misconduct did not experience a citation penalty in absolute terms (b = -0.58, t = -0.98, p = .325) or compared to their controls (b = -0.53, t = -0.73, p = .464). Despite the qualitatively larger citation penalty for those accused of sexual vs. scientific misconduct, the three-way interaction is not significant (b = -1.63, t = -1.69, p = .091).

- *Model 2 WHOLE DATASET* -
We regress average yearly citations per publication on Scholar Group (Control vs. Accused), Misconduct Type (Sexual vs. Scientific), Time (Pre- vs. Post-Accusations), number of authors (numeric), and their interactions, controlling for publication year, total citations per publication, scholar's discipline, rank, gender, the year the accusations became public, and time trend. This model reveals both a significant three-way interaction of interest (b = -5.47, t = -5.02, p < .001), showing that the citation penalty is larger for sexual vs. scientific misconduct, and a significant four-way interaction (b = 0.51, t = 4.46, p < .001).

- *Model 1 FEW AUTHORS DATASET* -
We regress average yearly citations per publication on Scholar Group (Control vs. Accused), Misconduct Type (Sexual vs. Scientific), Time (Pre- vs. Post-Accusations), and their interactions, controlling for publication year, total citations per publication, number of authors, scholar's discipline, rank, gender, the year the accusations became public, and time trend. The citation rates of scholars accused of sexual misconduct decreased both in absolute terms (b = -1.43, t = -2.90, p = .004) and compared to their controls (b = -2.65, t = -3.82, p < .001), indicating a citation penalty. However, this analysis indicates that scholars accused of scientific misconduct did not experience a citation penalty in absolute terms (b = -0.13, t = -0.23, p = .819) or compared to their controls (b = 0.64, t = 1.10, p = .273). Importantly, this model reveals that scholars accused of sexual misconduct received a *larger* citation penalty than those accused of scientific misconduct, shown by both a significant two-way interaction (b = -1.30, t = -2.28, p = .023) and by a significant three-way interaction (b = -3.29, t = -3.64, p < .001).

- *Model 3 ONLY ACCUSED OF SCIENTIFIC MISCONDUCT DATASET* -
We regress average yearly citations of researchers accused of scientific misconduct on Retraction Status (Yes vs. No), Time (Pre- vs. Post-accusation), and their interaction, controlling for publication year, total citations per publication, number of authors, scholar's discipline, rank, gender, the year the accusations became public, and time trend. The citations of retracted publications did not significantly decrease (b = -2.42, t = -1.49, p = .137), nor did the citations of non-retracted publications (b = -0.96, t = -1.28, p = .199). The citations of the retracted and non-retracted publications decreased to the same extent (b = -1.45, t = -0.96, p = .337).

REGRESSION ROBUSTNESS CHECKS

In this section we repeat our analysis on the whole dataset for Model 1 and Model 2, on the few-authors dataset for Model 1, and on the scientific accused only dataset for Model 3 across different regressions' specifications. Specifically, we ran each of our models using the following specifications:

- Including 'total citations per publication up to accusation year' (i.e., Controlling for 'Tot Cit Up To Accusation') as a covariate instead of 'total citations per publication' (i.e., pre- and post-accusation).
- Normalizing citations data by year within field (i.e., Normalization) instead of controlling for 'Publication Year', 'Field', and 'Time Trend'.

Regression Tables

Model 1: 2(Accusation Status: Control vs. Accused) x 2(Misconduct Type: Sexual vs. Scientific) x 2(Timing: Pre- vs. Post-Accusation) – WHOLE DATASET

	Average Yearly Citations per Publication	
	Controlling for 'Tot Cit Up To Accusation' (N=31,941) (Obs.=290,038)	Normalization by Year within Field (N=31,941) (Obs.=290,038)
Intercept	-261.441<br>(292.632)	0.036<br>(0.077)
Control	-0.333<br>(0.603)	-3.193×10^{-5}<br>(1.780×10^{-4})
Post-accusation	-2.144***<br>(0.449)	-3.682×10^{-4} ***<br>(8.757×10^{-5})
Scientific	-0.755<br>(1.184)	-4.653×10^{-4}<br>(3.119×10^{-4})
Control:Post-accusation	1.834***<br>(0.551)	1.180×10^{-4}<br>(9.957×10^{-5})
Control:Scientific	0.406<br>(0.839)	3.408×10^{-4}<br>(3.338×10^{-4})
Post-accusation:Scientific	1.272<br>(0.769)	2.204×10^{-4}<br>(1.200×10^{-4})
Control:Post-accusation:Scientific	-0.811<br>(1.092)	-2.869×10^{-4} *<br>(1.437×10^{-4})

Note: *p<0.05; **p<0.01; ***p<0.001

#Regressions include different covariates than the main analysis (see specifications above), but SEs are still clustered by Scholar in all regressions. See https://osf.io/ycazs/?view_only=bfb1080d756146ba89d21a7ed3daeacf for full regression code, data, and complete output.

Table 9. Regression table for Model 1 Whole Dataset – Regression robustness check[10]

[10] The relevant model descriptions and specifications are included in the following pages. (1) Controlling for 'Tot Cit Up To Accusation': page 28, (2) Normalization by Year within Field: page 29.

Model 2: Adding 'AuthorCount' as an interaction term – WHOLE DATASET

	Average Yearly Citations per Publication	
	Controlling for 'Tot Cit Up To Accusation' (N=31,941) (Obs.=290,038)	Normalization by Year within Field (N=31,941) (Obs.=290,038)
Intercept	-72.291 (266.701)	0.026 (0.075)
Control	0.610 (0.481)	-1.782×10^{-4} (1.771×10^{-4})
Post-accusation	-1.718* (0.680)	-3.657×10^{-4} *** (9.313×10^{-5})
Scientific	-1.879 (1.475)	-2.411×10^{-4} (2.867×10^{-4})
AuthorCount	0.215*** (0.054)	-4.260×10^{-6} (8.225×10^{-6})
Control:Post-accusation	2.069** (0.739)	2.268×10^{-4} * (1.119×10^{-4})
Control:Scientific	-0.489 (1.265)	2.891×10^{-4} (3.161×10^{-4})
Post-accusation:Scientific	1.853 (1.007)	1.940×10^{-4} (1.418×10^{-4})
Control:AuthorCount	-0.132* (0.059)	2.060×10^{-5} (1.540×10^{-5})
Post-accusation:AuthorCount	-0.073 (0.074)	5.310×10^{-7} (1.505×10^{-6})
Scientific:AuthorCount	0.143 (0.152)	-3.759×10^{-5} (3.075×10^{-5})
Control:Post-accusation:Scientific	-5.490*** (1.130)	-3.629×10^{-4} * (1.718×10^{-4})
Control:Post-accusation:AuthorCount	0.040 (0.077)	-1.462×10^{-5} (1.158×10^{-5})
Control:Scientific:AuthorCount	0.104 (0.160)	1.367×10^{-5} (3.531×10^{-5})
Post-accusation:Scientific:AuthorCount	-0.114 (0.096)	5.870×10^{-6} (1.864×10^{-5})
Control:Post-accusation:Scientific:AuthorCount	0.577*** (0.101)	9.749×10^{-6} (2.352×10^{-5})

Note: *p<0.05; **p<0.01; ***p<0.001

Regressions include different covariates than the main analysis (see specifications above), but SEs are still clustered by Scholar in all regressions. See https://osf.io/ycazs/?view_only=bfb1080d756146ba89d21a7ed3daeacf for full regression code, data, and complete output.

Table 10. Regression table for Model 2 Whole Dataset – Regression robustness check[11]

[11] The relevant model descriptions and specifications are included in the following pages. (1) Controlling for 'Tot Cit Up To Accusation': page 28, (2) Normalization by Year within Field: page 29.

Model 1: 2(Accusation Status: Control vs. Accused) x 2(Misconduct Type: Sexual vs. Scientific) x 2(Timing: Pre- vs. Post-Accusation) – FEW AUTHORS DATASET

	Average Yearly Citations per Publication	
	Controlling for 'Tot Cit Up To Accusation' (N=23,911) (Obs.=229,042)	Normalization by Year within Field (N=23,911) (Obs.=229,042)
Intercept	-168.179 (215.498)	0.005 (0.073)
Control	0.059 (0.558)	-1.697×10^{-4} (1.656×10^{-4})
Post-accusation	-1.949*** (0.440)	-3.655×10^{-4} *** (9.293×10^{-5})
Scientific	-1.467 (1.026)	-2.573×10^{-4} (2.711×10^{-4})
Control:Post-accusation	2.456*** (0.593)	1.981×10^{-4} * (9.950×10^{-5})
Control:Scientific	-0.051 (0.823)	4.366×10^{-4} (2.908×10^{-4})
Post-accusation:Scientific	1.649* (0.742)	2.179×10^{-4} (1.215×10^{-4})
Control:Post-accusation:Scientific	-3.049*** (0.924)	-3.563×10^{-4} * (1.441×10^{-4})

Note: *p<0.05; **p<0.01; ***p<0.001

Regressions include different covariates than the main analysis (see specifications above), but SEs are still clustered by Scholar in all regressions. See https://osf.io/ycazs/?view_only=bfb1080d756146ba89d21a7ed3daeacf for full regression code, data, and complete output.

Table 11. Regression table for Model 1 Few Authors Dataset – Regression robustness check[12]

Model 3: 2(Retraction Status: No vs. Yes) x 2(Timing: Pre- vs. Post-Accusation) – SCIENTIFIC ACCUSED ONLY WHOLE DATASET

	Average Yearly Citations per Publication	
	Controlling for 'Tot Cit Up To Accusation' (N=3,056) (Obs.=28,228)	Normalization by Year within Field (N=3,056) (Obs.=28,228)
Intercept	-347.694 (830.484)	0.116 (0.140)
Post-accusation	-3.679* (1.795)	-1.067×10^{-3} (6.985×10^{-4})
NoRetraction	0.167 (1.528)	-8.935×10^{-4} (7.384×10^{-4})
Post-accusation:NoRetraction	1.580 (1.381)	9.069×10^{-4} (6.842×10^{-4})

Note: *p<0.05; **p<0.01; ***p<0.001

Regressions include different covariates than the main analysis (see specifications above), but SEs are still clustered by Scholar in all regressions. See https://osf.io/ycazs/?view_only=bfb1080d756146ba89d21a7ed3daeacf for full regression code, data, and complete output.

Table 12. Regression table for Model 3 Scientific Accused Only Dataset – Regression robustness check[13]

[12] The relevant model descriptions and specifications are included in the following pages. (1) Controlling for 'Tot Cit Up To Accusation': page 28, (2) Normalization by Year within Field: page 29.

[13] The relevant model descriptions and specifications are included in the following pages. (1) Controlling for 'Tot Cit Up To Accusation': page 28, (2) Normalization by Year within Field: page 29.

Controlling for 'Total citations per publication up to accusation year' instead of 'total citations per publication'

- *Model 1 WHOLE DATASET* -
We regress average yearly citations per publication on Scholar Group (Control vs. Accused), Misconduct Type (Sexual vs. Scientific), Time (Pre- vs. Post-Accusations), and their interactions, controlling for publication year, total citations per publication up to accusation, number of authors, scholar's discipline, rank, gender, the year the accusations became public, and time trend. The citation rates of scholars accused of sexual misconduct decreased both in absolute terms (b = -2.14, t = -4.78, p < .001) and compared to their controls (b = -1.83, t = -3.33, p < .001), indicating a citation penalty. However, this analysis indicates that scholars accused of scientific misconduct did not experience a citation penalty in absolute terms (b = -0.87, t = -1.30, p = .194) or compared to their controls (b = -1.02, t = -1.13, p = .257). Despite the qualitatively larger citation penalty for those accused of sexual vs. scientific misconduct, the three-way interaction is not significant (b = -0.81, t = -0.74, p = .457).

- *Model 2 WHOLE DATASET* -
We regress average yearly citations per publication on Scholar Group (Control vs. Accused), Misconduct Type (Sexual vs. Scientific), Time (Pre- vs. Post-Accusations), number of authors (numeric), and their interactions, controlling for publication year, total citations per publication up to accusation, scholar's discipline, rank, gender, the year the accusations became public, and time trend. This model reveals both a significant three-way interaction of interest (b = -5.49, t = -4.86, p < .001), showing that the citation penalty is larger for sexual vs. scientific misconduct, and a significant four-way interaction (b = 0.58, t = 5.74, p < .001).

- *Model 1 FEW AUTHORS DATASET* -
We regress average yearly citations per publication on Scholar Group (Control vs. Accused), Misconduct Type (Sexual vs. Scientific), Time (Pre- vs. Post-Accusations), and their interactions, controlling for publication year, total citations per publication up to accusation, number of authors, scholar's discipline, rank, gender, the year the accusations became public, and time trend. The citation rates of scholars accused of sexual misconduct decreased both in absolute terms (b = -1.95, t = -4.43, p < .001) and compared to their controls (b = -2.46, t = -4.14, p < .001), indicating a citation penalty. However, this analysis indicates that scholars accused of scientific misconduct did not experience a citation penalty in absolute terms (b = -0.30, t = -0.45, p = .649) or compared to their controls (b = -0.59, t = -0.82, p = .411). Importantly, this model reveals that scholars accused of sexual misconduct received a *larger* citation penalty than those accused of scientific misconduct, shown by a significant two-way interaction (b = -1.65, t = -2.22, p = .026) and by a significant three-way interaction (b = -3.05, t = -3.30, p < .001).

- *Model 3 ONLY ACCUSED OF SCIENTIFIC MISCONDUCT DATASET* -
We regress average yearly citations of researchers accused of scientific misconduct on Retraction Status (Yes vs. No), Time (Pre- vs. Post-accusation), and their interaction, controlling for publication year, total citations per publication up to accusation, number of authors, scholar's discipline, rank, gender, the year the accusations became public, and time trend. The citations of retracted publications significantly decreased (b = -3.68, t = -2.05, p = .040), while the citations of non-retracted publications did not (b = -2.10, t = -1.59, p = .112). The citations of the retracted and non-retracted publications decreased to the same extent (b = -1.58, t = -1.14, p = .253).

Normalizing citations data by year within field

- *Model 1 WHOLE DATASET* -
We regress average yearly citations per publication normalized by year within scholar's discipline on Scholar Group (Control vs. Accused), Misconduct Type (Sexual vs. Scientific), Time (Pre- vs. Post-Accusations), and their interactions, controlling for total citations per publication, number of authors, scholar's rank, gender, and the year the accusations became public. The citation rates of scholars accused of sexual misconduct decreased in absolute terms (b = -3.68x10^{-4}, t = -4.21, p < .001) but not compared to their controls (b = -1.18x10^{-4}, t = -1.19, p = .236). This analysis also indicates that scholars accused of scientific misconduct experienced a marginal citation penalty in absolute terms (b = -1.48x10^{-4}, t = -1.83, p = .067) and no penalty compared to their controls (b = 1.69x10^{-4}, t = 1.64, p = .101). Importantly, this model reveals that scholars accused of sexual misconduct received a *larger* citation penalty than those accused of scientific misconduct, shown by a marginally significant two-way interaction (b = -2.20x10^{-4}, t = -1.84, p = .066) and by a significant three-way interaction (b = -2.87x10^{-4}, t = -2.00, p = .046).

- *Model 2 WHOLE DATASET* -
We regress average yearly citations per publication normalized by year within scholar's discipline on Scholar Group (Control vs. Accused), Misconduct Type (Sexual vs. Scientific), Time (Pre- vs. Post-Accusations), number of authors (numeric), and their interactions, controlling for total citations per publication, number of authors, scholar's rank, gender, and the year the accusations became public. This model reveals a significant three-way interaction of interest (b = -3.63x10^{-4}, t = -2.11, p = .035), showing that the citation penalty is *larger* for sexual vs. scientific misconduct, but fails to show a significant four-way interaction (b = 9.75x10^{-6}, t = 0.41, p = .679).

- *Model 1 FEW AUTHORS DATASET* -
We regress average yearly citations per publication normalized by year within scholar's discipline on Scholar Group (Control vs. Accused), Misconduct Type (Sexual vs. Scientific), Time (Pre- vs. Post-Accusations), and their interactions, controlling for total citations per publication, number of authors, scholar's rank, gender, and the year the accusations became public. The citation rates of scholars accused of sexual misconduct decreased both in absolute terms (b = -3.65x10^{-4}, t = -3.93, p < .001) and compared to their controls (b = -1.98x10^{-4}, t = -1.99, p = .046), indicating a citation penalty. However, this analysis indicates that scholars accused of scientific misconduct experienced only a marginal citation penalty in absolute terms (b = -1.48x10^{-4}, t = -1.91, p = .056) and no penalty compared to their controls (b = 1.58x10^{-4}, t = 1.55, p = .120). Importantly, this model reveals that scholars accused of sexual misconduct received a *larger* citation penalty than those accused of scientific misconduct, shown by a marginally significant two-way interaction (b = -2.18x10^{-4}, t = -1.79, p = .073) and by a significant three-way interaction (b = -3.56x10^{-4}, t = -2.47, p = .013).

- *Model 3 ONLY ACCUSED OF SCIENTIFIC MISCONDUCT DATASET* -
We regress average yearly citations per publication of researchers accused of scientific misconduct normalized by year within scholar's discipline on Retraction Status (Yes vs. No), Time (Pre- vs. Post-accusation), and their interaction, controlling for total citations per publication, number of authors, scholar's rank, gender, and the year the accusations became public. The citations of retracted publications did not significantly decrease (b = -1.07x10^{-3}, t = -1.53, p = .127), while the citations of non-retracted publications did significantly decrease (b = -1.60x10^{-4}, t = -2.94, p = .003). The citations of the retracted and non-retracted publications, however, decreased to the same extent (b = -9.07x10^{-4}, t = -1.33, p = .185).

Note that we included this analysis here as a robustness check (as opposed to as the main analysis in the manuscript), because this analysis has an important limitation within the scope of our research. Specifically, the normalization herein was performed on our relatively limited dataset. In contrast, normalization in the literature is typically conducted on massive datasets with extremely high numbers of publications in a given field, providing a more robust controlling mechanism.

CONTROL SCHOLARS ROBUSTNESS CHECK

In this section we conceptually replicate our main analyses (Model 1 in the paper) across different controls' specifications. Specifically, we ran the following analyses:

- Excluding control scholars.
- Merging our two control scholars' groups into a single control group, therefore comparing the citations of only three groups of scholars (i.e., scholars accused of sexual misconduct, scholars accused of scientific misconduct, and control scholars) before and after the accusations became public.

Finally, we ran a cross validation analysis on the control scholars (leave-one-out cross validation; LOO-CV) and we find that our results hold when dropping any control scholar.

Why we do not use Propensity Score Matching or Synthetic Controls

We would like to highlight that in this paper we did not analyze an existing dataset, but instead built the dataset ourselves by manually identifying, collecting, and organizing Web of Science citation data for our scholars of interest. In other words, we had to do the matching before collecting the data. For this reason, we could not use propensity score matching (PSM) or synthetic controls (SC) for controls matching.

<u>Propensity Score Matching</u>: PSM offers a rigorous method for estimating treatment effects by matching units with similar characteristics across treatment and control groups. To properly implement PSM in our investigation, we would need to assemble a comprehensive dataset encompassing a wide range of scholars, virtually extending into all scholars in the 18 fields our accused belong to. This dataset would then require the creation of precise and objective qualitative assessments for each scholar to facilitate the algorithm's ability to identify optimal controls. However, no such dataset exists, and repositories like Web of Science and Google Scholar have strict legal policies that restrict data scraping. Given this, the implementation of PSM in our study was not feasible, compelling us to explore alternative methodologies to address our research objectives.

<u>Synthetic Controls</u>: Similar to the case of PSM above, implementing SC for our investigation would require access to an extensive dataset that includes a wide range of scholars that would then allow us to identify appropriate control scholars with similar pre-accusation citation trends, essential for constructing accurate synthetic controls (Abadie, 2021). Consequently, without the necessary foundational data to accurately model pre-accusation citation trends, the application of the Synthetic Controls method was not a viable option, prompting us to explore alternative approaches to address our research question, within the context of the constraints and data availability issues we encountered.

Regression Tables

Model 4: 2(Misconduct Type: Sexual vs. Scientific) x 2(Timing: Pre- vs. Post-Accusation) – ACCUSED ONLY WHOLE DATASET & Model 5: 3(Scholar Group: Sexual Accused vs. Scientific Accused vs. Control) x 2(Timing: Pre- vs. Post-Accusation) – WHOLE DATASET

	Average Yearly Citations per Publication (Reference: Sexual Accused)	
	No controls (N=5,888) (Obs.=56,645)	Single control group (N=31,941) (Obs.=290,038)
Intercept	360.397 (278.178)	-360.064** (138.863)
Control		-0.970* (0.377)
Scientific	-3.027*** (0.656)	-1.067 (0.623)
Post-accusation	-1.786* (0.830)	-1.730*** (0.450)
Post-accusation:Control		1.847*** (0.466)
Post-accusation:Scientific	0.777 (0.605)	0.920 (0.616)

Note: *p<0.05; **p<0.01; ***p<0.001

	Average Yearly Citations per Publication (Reference: Scientific Accused)	
	No controls (N=5,888) (Obs.=56,645)	Single control group (N=31,941) (Obs.=290,038)
Intercept	357.370 (277.631)	-361.131** (138.761)
Control		0.096 (0.501)
Sexual	3.027*** (0.656)	1.067 (0.623)
Post-accusation	-1.008 (0.784)	-0.809 (0.545)
Post-accusation:Control		0.927 (0.605)
Post-accusation:Sexual	-0.777 (0.605)	-0.920 (0.616)

Note: *p<0.05; **p<0.01; ***p<0.001

#Regression includes controls for the same covariates as the main analysis (i.e., publication year, number of authors, total citations per publication, scholar's discipline, rank, gender, the year the accusations became public, and time trend), and SEs clustered by Scholar. See https://osf.io/ycazs/?view_only=bfb1080d756146ba89d21a7ed3daeacf for full regression code, data, and complete output.

Table 13. Regression table for main results on Whole Datasets – Controls robustness check[14]

[14] The relevant model descriptions and specifications are included at page 33.

Model 4: 2(Misconduct Type: Sexual vs. Scientific) x 2(Timing: Pre- vs. Post-Accusation) – ACCUSED ONLY FEW AUTHORS DATASET & Model 5: 3(Scholar Group: Sexual Accused vs. Scientific Accused vs. Control) x 2(Timing: Pre- vs. Post-Accusation) – FEW AUTHORS DATASET

	Average Yearly Citations per Publication (Reference: Sexual Accused)	
	No controls (N=4,532) (Obs.=44,598)	Single control group (N=23,911) (Obs.=229,042)
Intercept	368.935<br>(319.638)	-248.179<br>(129.802)
Control		-0.546<br>(0.374)
Scientific	-3.503***<br>(0.658)	-0.606<br>(0.640)
Post-accusation	-1.037*<br>(0.422)	-1.605***<br>(0.450)
Post-accusation:Control		1.847***<br>(0.465)
Post-accusation:Scientific	1.109*<br>(0.562)	1.273*<br>(0.590)

Note: *p<0.05; **p<0.01; ***p<0.001

	Average Yearly Citations per Publication (Reference: Scientific Accused)	
	No controls (N=4,532) (Obs.=44,598)	Single control group (N=23,911) (Obs.=229,042)
Intercept	365.432<br>(319.046)	-248.785<br>(129.644)
Control		0.060<br>(0.568)
Sexual	3.503***<br>(0.658)	0.606<br>(0.640)
Post-accusation	0.072<br>(0.391)	-0.333<br>(0.450)
Post-accusation:Control		0.358<br>(0.566)
Post-accusation:Sexual	-1.109*<br>(0.562)	-1.273*<br>(0.590)

Note: *p<0.05; **p<0.01; ***p<0.001

#Regression includes controls for the same covariates as the main analysis (i.e., publication year, number of authors, total citations per publication, scholar's discipline, rank, gender, the year the accusations became public, and time trend), and SEs clustered by Scholar. See https://osf.io/ycazs/?view_only=bfb1080d756146ba89d21a7ed3daeacf for full regression code, data, and complete output.

Table 14. Regression table for main results on Few Authors Datasets – Controls robustness check[15]

[15] The relevant model descriptions and specifications are included at page 33.

Control Scholars Robustness Checks

- *Model 4 ONLY ACCUSED WHOLE DATASET* -
We regress average yearly citations per publication of accused scholars on Misconduct Type (Sexual vs. Scientific), Time (Pre- vs. Post-Accusations), and their interaction, controlling for publication year, total citations per publication, number of authors, scholar's discipline, rank, gender, the year the accusations became public, and time trend. The citation rates of scholars accused of sexual misconduct decreased (b = -1.79, t = -2.15, p = .032), indicating a citation penalty. However, this analysis indicates that scholars accused of scientific misconduct did not experience a citation penalty (b = -1.01, t = -1.29, p = .199). Despite the qualitatively larger citation penalty for those accused of sexual vs. scientific misconduct, the interaction is not significant (b = -0.78, t = -1.28, p = .199).

- *Model 4 ONLY ACCUSED FEW AUTHORS DATASET* -
We regress average yearly citations per publication of accused scholars on Misconduct Type (Sexual vs. Scientific), Time (Pre- vs. Post-Accusations), and their interaction, controlling for publication year, total citations per publication, number of authors, scholar's discipline, rank, gender, the year the accusations became public, and time trend. The citation rates of scholars accused of sexual misconduct decreased (b = -1.04, t = -2.46, p = .014), indicating a citation penalty. However, this analysis indicates that scholars accused of scientific misconduct did not experience a citation penalty (b = 0.07, t = 0.18, p = .853). Importantly, this model reveals that scholars accused of sexual misconduct received a *larger* citation penalty than those accused of scientific misconduct, shown by a significant two-way interaction (b = -1.11, t = -1.98, p = .048).

- *Model 5 WHOLE DATASET* -
We regress average yearly citations per publication on Scholar Group (Control vs. Accused Sexual vs. Accused Scientific), Time (Pre- vs. Post-Accusations), and their interactions, controlling for publication year, total citations per publication, scholar's discipline, rank, gender, the year the accusations became public, and time trend. The citation rates of scholars accused of sexual misconduct decreased (b = -1.73, t = -3.84, p < .001), indicating a citation penalty. However, this analysis indicates that scholars accused of scientific misconduct did not experience a citation penalty (b = -0.81, t = -1.48, p = .138), nor did the controls (b = 0.12, t = 0.42, p = .678). This model reveals that scholars accused of sexual misconduct received a larger citation penalty than controls (b = -1.85, t = -3.96, p < .001), but as large as those accused of scientific misconduct (b = -0.92, t = -1.49, p = .135). Scholars accused of scientific misconduct and controls were affected to same extent (b = -0.93, t = -1.53, p = .126).

- *Model 5 FEW AUTHORS DATASET* -
We regress average yearly citations per publication on Scholar Group (Control vs. Accused Sexual vs. Accused Scientific), Time (Pre- vs. Post-Accusations), and their interactions, controlling for publication year, total citations per publication, scholar's discipline, rank, gender, the year the accusations became public, and time trend. The citation rates of scholars accused of sexual misconduct decreased (b = -1.61, t = -3.45, p < .001), indicating a citation penalty. However, this analysis indicates that scholars accused of scientific misconduct did not experience a citation penalty (b = -0.33, t = -0.65, p = .518), nor did the controls (b = 0.02, t = 0.17, p = .866). This model reveals that scholars accused of sexual misconduct received a *larger* citation penalty than controls (b = -1.63, t = -3.28, p = .001), and *larger* than those accused of scientific misconduct (b = -1.27, t = -2.16, p = .031). Scholars accused of scientific misconduct and controls were affected to same extent (b = -0.36, t = -0.63, p = .528).

***Leave One Out – Cross Validation (LOO-CV) analysis on control scholars** - Model 1 WHOLE DATASET*

	Average Yearly Citations per Publication (Reference: Accused, Sexual)					
	Mean Estimate	SD Estimate	Mean SE	Mean t-value	Mean p-value	% Significant
Intercept	-202.90	22.24	176.44	-1.15	0.25	0.70
Control	-0.92	0.03	0.38	-2.43	0.02	100.00
Post-accusation	-1.74	0.02	0.45	-3.85	0.00	100.00
Scientific	-2.27	0.07	0.86	-2.64	0.01	100.00
Control:Post-accusation	2.05	0.04	0.56	3.64	0.00	100.00
Control:Scientific	0.87	0.04	0.59	1.47	0.14	0.00
Post-accusation:Scientific	0.92	0.01	0.62	1.49	0.14	0.00
Control:Post-accusation:Scientific	-1.31	0.06	0.91	-1.44	0.15	0.70

	Average Yearly Citations per Publication (Reference: Accused, Scientific)					
	Mean Estimate	SD Estimate	Mean SE	Mean t-value	Mean p-value	% Significant
Intercept	-205.17	22.19	175.99	-1.17	0.25	0.70
Control	-0.05	0.03	0.45	-0.11	0.90	0.00
Post-accusation	-0.82	0.02	0.55	-1.51	0.13	0.00
Sexual	2.27	0.07	0.86	2.64	0.01	100.00
Control:Post-accusation	0.74	0.04	0.70	1.06	0.29	0.00
Control: Sexual	-0.87	0.04	0.59	-1.47	0.14	0.00
Post-accusation: Sexual	-0.92	0.01	0.62	-1.49	0.14	0.00
Control:Post-accusation: Sexual	1.31	0.06	0.91	1.44	0.15	0.70

	Average Yearly Citations per Publication (Reference: Control, Sexual)					
	Mean Estimate	SD Estimate	Mean SE	Mean t-value	Mean p-value	% Significant
Intercept	-203.83	22.25	176.46	-1.16	0.25	0.70
Accused	0.92	0.03	0.38	2.43	0.02	100.00
Post-accusation	0.31	0.03	0.36	0.85	0.40	0.00
Scientific	-1.39	0.08	0.84	-1.65	0.10	1.41
Accused:Post-accusation	-2.05	0.04	0.56	-3.64	0.00	100.00
Accused:Scientific	-0.87	0.04	0.59	-1.47	0.14	0.00
Post-accusation:Scientific	-0.39	0.06	0.68	-0.57	0.57	0.00
Accused:Post-accusation:Scientific	1.31	0.06	0.91	1.44	0.15	0.70

	Average Yearly Citations per Publication (Reference: Control, Scientific)					
	Mean Estimate	SD Estimate	Mean SE	Mean t-value	Mean p-value	% Significant
Intercept	-205.22	22.19	175.98	-1.17	0.25	0.70
Accused	0.05	0.03	0.45	0.11	0.90	0.00
Post-accusation	-0.08	0.04	0.52	-0.16	0.87	0.00
Sexual	1.39	0.08	0.84	1.65	0.10	1.41
Accused:Post-accusation	-0.74	0.04	0.70	-1.06	0.29	0.00
Accused: Sexual	0.87	0.04	0.59	1.47	0.14	0.00
Post-accusation: Sexual	0.39	0.06	0.68	0.57	0.57	0.00
Accused:Post-accusation: Sexual	-1.31	0.06	0.91	-1.44	0.15	0.70

Leave One Out – Cross Validation (LOO-CV) analysis on control scholars - *Model 2 WHOLE DATASET*

	Average Yearly Citations per Publication (Reference: Accused, Sexual)					
	Mean Estimate	SD Estimate	Mean SE	Mean t-value	Mean p-value	% Significant
Intercept	-75.93	21.72	161.83	-0.47	0.63	0.00
Control	-0.22	0.03	0.45	-0.49	0.63	0.00
Post-accusation	-1.24	0.02	0.70	-1.78	0.08	0.07
Scientific	-2.54	0.07	1.16	-2.19	0.03	99.30
AuthorCount	0.18	0.00	0.05	3.64	0.00	100.00
Control:Post-accusation	2.08	0.05	0.78	2.65	0.01	100.00
Control:Scientific	1.06	0.05	1.02	1.05	0.30	0.00
Post-accusation:Scientific	1.43	0.01	0.85	1.69	0.09	0.00
Control:AuthorCount	-0.11	0.01	0.06	-1.91	0.06	2.82
Post-accusation:AuthorCount	-0.09	0.00	0.07	-1.20	0.23	0.00
Scientific:AuthorCount	0.03	0.00	0.11	0.28	0.78	0.00
Control:Post-accusation:Scientific	-5.17	0.07	1.07	-4.84	0.00	100.00
Control:Post-accusation:AuthorCount	0.04	0.00	0.08	0.57	0.00	0.00
Control:Scientific:AuthorCount	-0.06	0.01	0.12	-0.53	0.60	0.00
Post-accusation:Scientific:AuthorCount	-0.09	0.00	0.09	-1.01	0.31	0.00
Control:Post-accusation:Scientific:AuthorCount	0.53	0.01	0.11	4.90	0.00	100.00

Leave One Out – Cross Validation (LOO-CV) analysis on control scholars - *Model 1 FEW AUTHORS DATASET*

	Average Yearly Citations per Publication (Reference: Accused, Sexual)					
	Mean Estimate	SD Estimate	Mean SE	Mean t-value	Mean p-value	% Significant
Intercept	25.99	18.13	151.38	0.17	0.85	0.00
Control	-0.62	0.02	0.38	-1.61	0.11	0.00
Post-accusation	-1.62	0.03	0.46	-3.50	0.00	100.00
Scientific	-2.55	0.06	0.76	-3.37	0.00	100.00
Control:Post-accusation	2.45	0.04	0.64	3.85	0.00	100.00
Control:Scientific	0.63	0.03	0.60	1.05	0.29	0.00
Post-accusation:Scientific	1.27	0.00	0.58	2.19	0.03	100.00
Control:Post-accusation:Scientific	-2.91	0.05	0.83	-3.48	0.00	100.00

	Average Yearly Citations per Publication (Reference: Accused, Scientific)					
	Mean Estimate	SD Estimate	Mean SE	Mean t-value	Mean p-value	% Significant
Intercept	23.44	18.08	150.96	0.15	0.87	0.00
Control	0.01	0.03	0.48	0.02	0.97	0.00
Post-accusation	-0.35	0.03	0.51	-0.69	0.49	0.00
Sexual	2.55	0.06	0.76	3.37	0.00	100.00
Control:Post-accusation	-0.46	0.03	0.55	-0.83	0.41	0.00
Control: Sexual	-0.63	0.03	0.60	-1.05	0.29	0.00
Post-accusation: Sexual	-1.27	0.00	0.58	-2.19	0.03	100.00
Control:Post-accusation: Sexual	2.91	0.05	0.83	3.48	0.00	100.00

	Average Yearly Citations per Publication (Reference: Control, Sexual)					
	Mean Estimate	SD Estimate	Mean SE	Mean t-value	Mean p-value	% Significant
Intercept	25.37	18.13	151.37	0.17	0.86	0.00
Accused	0.62	0.02	0.38	1.61	0.11	0.00
Post-accusation	0.83	0.03	0.36	2.33	0.02	100.00
Scientific	-1.92	0.07	0.71	-2.70	0.01	100.00
Accused:Post-accusation	-2.45	0.04	0.64	-3.85	0.00	100.00
Accused:Scientific	-0.63	0.03	0.60	-1.05	0.29	0.00
Post-accusation:Scientific	-1.63	0.05	0.60	-2.72	0.01	100.00
Accused:Post-accusation:Scientific	2.91	0.05	0.83	3.48	0.00	100.00

	Average Yearly Citations per Publication (Reference: Control, Scientific)					
	Mean Estimate	SD Estimate	Mean SE	Mean t-value	Mean p-value	% Significant
Intercept	23.45	18.08	150.96	0.15	0.87	0.00
Accused	-0.01	0.03	0.48	-0.02	0.97	0.00
Post-accusation	-0.81	0.03	0.34	-2.40	0.02	100.00
Sexual	1.92	0.07	0.71	2.70	0.01	100.00
Accused:Post-accusation	0.46	0.03	0.55	0.83	0.41	0.00
Accused: Sexual	0.63	0.03	0.60	1.05	0.29	0.00
Post-accusation: Sexual	1.63	0.05	0.60	2.72	0.01	100.00
Accused:Post-accusation: Sexual	-2.91	0.05	0.83	-3.48	0.00	100.00

'NON-ACADEMICS' STUDY[16]

Material

In the survey we first defined scientific and sexual misconduct in academia.

Definitions

Academic institutions such as colleges and universities have been experiencing an increase in the number of complaints involving either scientific misconduct or sexual misconduct.

In academia, **scientific misconduct** includes cases where a researcher violates the standard ethical research practices in the publication of scientific research. Examples include data fabrication and the publication of false scientific results, which biases scientific knowledge and might even impact real outcomes, like misleading companies into adopting suboptimal advertising strategies, or misleading doctors into adopting suboptimal treatments for sick patients.

In academia, **sexual misconduct** includes cases where a teacher or professor engages in unwelcome actions of a sexual nature with a student or another faculty of lower rank. Examples include a professor demanding sexual favors from a student in exchange for good grades, or a supervisor persistently making suggestive remarks and/or giving unwanted verbal or physical attention towards a direct subordinate to the point where this creates a hostile work environment.

When answering the questions below, please remember that each type of misconduct can vary on several dimensions, such as the extent to which they negatively impact others or the extent to which the behavior is indicative of a pattern versus being a on-off incident.

Fig. 1. Definition of scientific and sexual misconduct in academia given in the survey.

On a separate page, we asked participants to indicate what types of misconduct (randomized) they just read about

What types of misconduct did we just describe? (pick two)

- ☐ Sexual
- ☐ Scientific
- ☐ Tax
- ☐ Driving
- ☐ Social
- ☐ Professional
- ☐ Financial

Fig. 2. Attention check question in the survey.

---

[16] Data, Material (qsf file), and R code for the analysis can be found on https://osf.io/ycazs/?view_only=bfb1080d756146ba89d21a7ed3daeacf .

Then, we asked participants to indicate which of the two types of misconduct is (a) more deserving of punishment, (b) more disgusting, and (c) worse than the other.

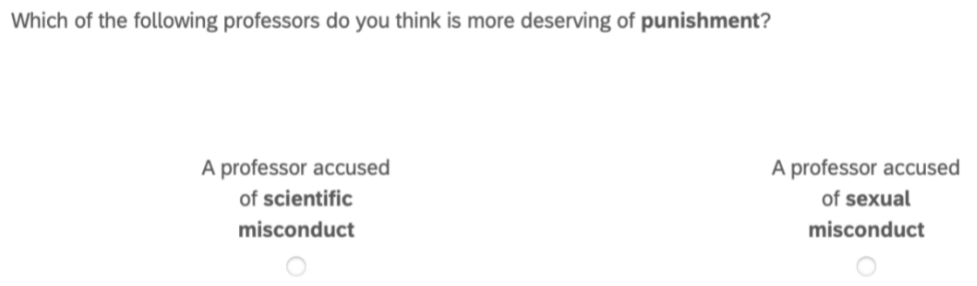

Fig. 3. Punishment question in the survey.

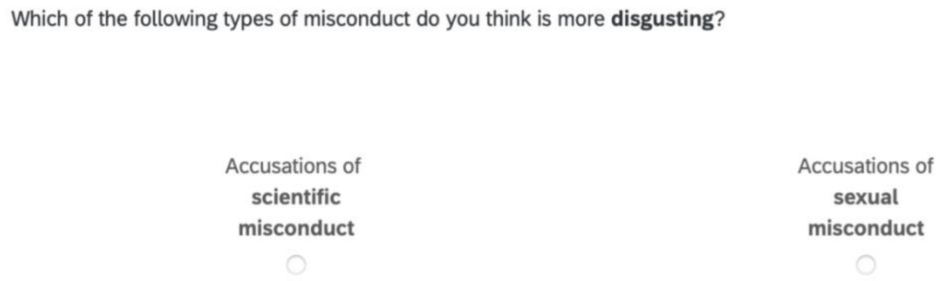

Fig. 4. Disgust question in the survey.

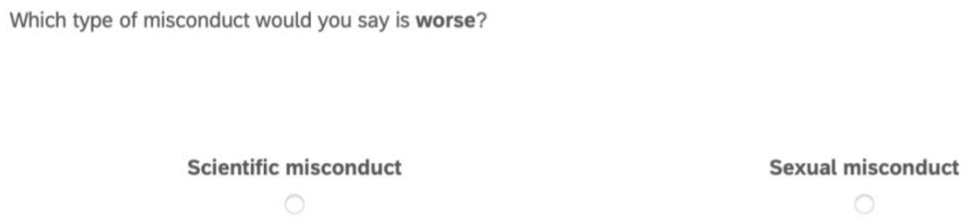

Fig. 5. Which misconduct type is worse question in the survey.

Note that, while the answer options' order within each question was randomized, these three questions' order was *not* randomized.

Results' Graphs

Analysis on overall sample (N=231)

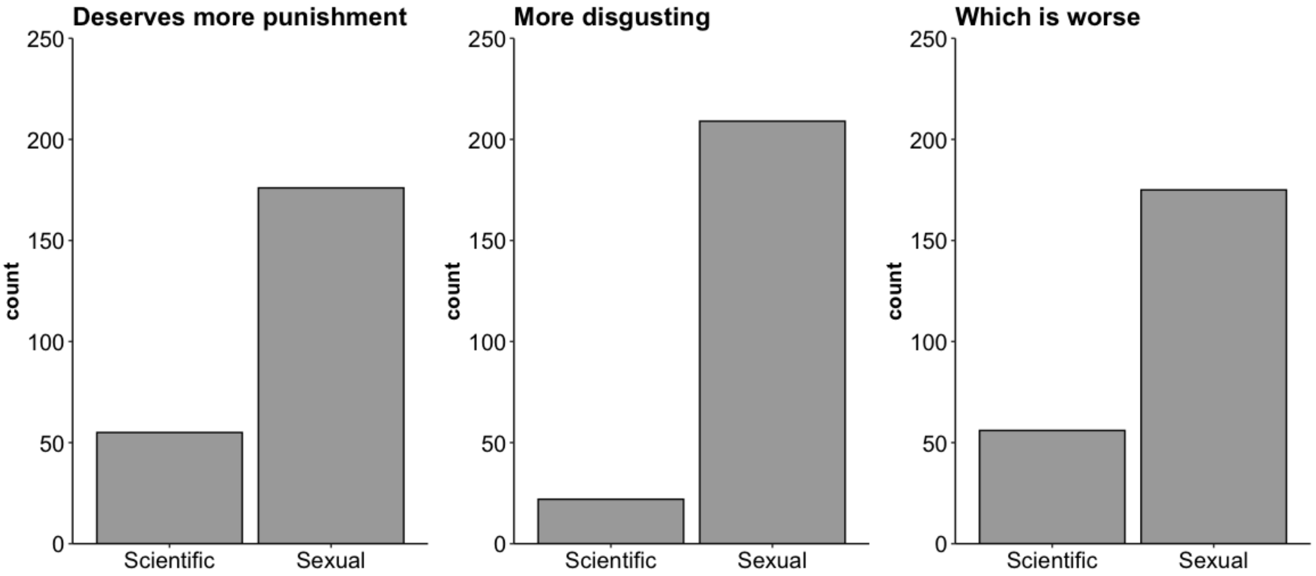

Fig. 6. Results on overall survey sample.

Analysis by Gender

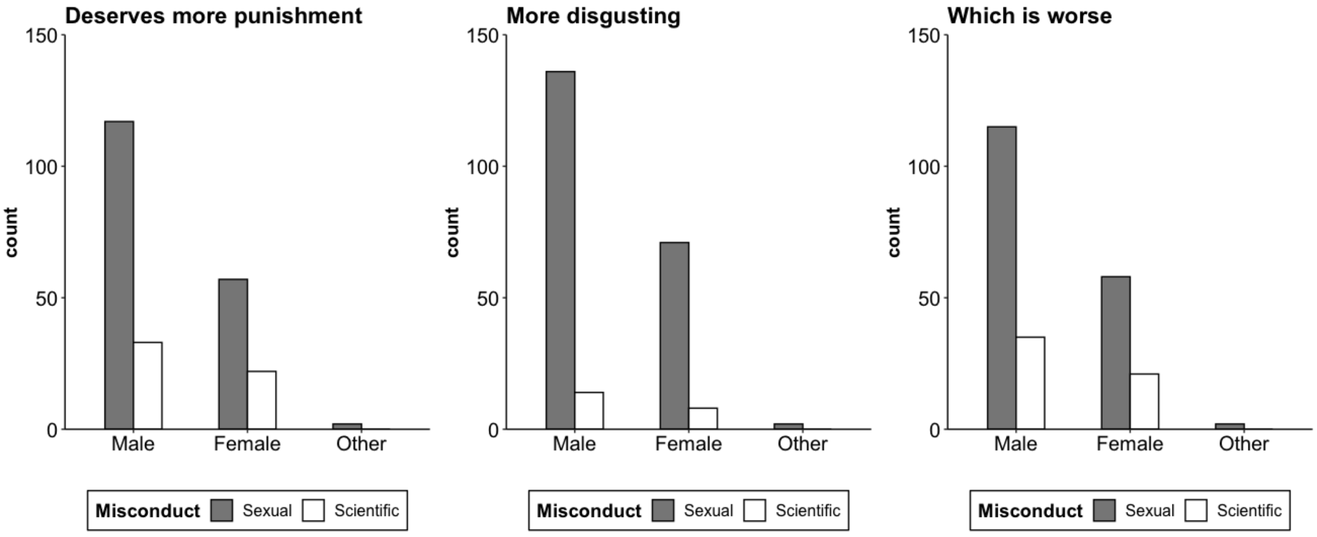

Fig. 7. Results by gender from the survey.

‘ACADEMICS’ STUDY[17]

Material

In the survey we first defined scientific and sexual misconduct in academia.

Definitions

Here below are the definitions of scientific misconduct and sexual misconduct in academia.

Scientific misconduct refers to cases where a researcher violates the standard ethical research practices in the publication of scientific research. Examples include data fabrication and the publication of false scientific results. Scientific misconduct biases scientific knowledge and might even impact real outcomes, like misleading companies into adopting suboptimal advertising strategies, or misleading doctors into adopting suboptimal treatments for sick patients.

Sexual misconduct refers to cases where a faculty member engages in unwelcome actions of a sexual nature with a student or another faculty of lower rank. Examples include a professor demanding sexual favors from a student in exchange for good grades or engaging in unwanted verbal or physical attention toward a lower-rank faculty. Sexual misconduct undermines the target(s)' well-being and creates a hostile work environment.

When answering the following questions, please remember that the negative impact of each type of misconduct may vary dramatically on several dimensions, such as the extent to which it negatively impacts others or the extent to which the behavior is indicative of a pattern versus being a one-time incident.

Fig. 8. Definition of scientific and sexual misconduct in academia given in the survey.

[17] Data, Material (qsf file), and R code for the analysis can be found on https://osf.io/ycazs/?view_only=bfb1080d756146ba89d21a7ed3daeacf .

Then, we asked participants to indicate how likely they would be to cite an article published by a scholar accused of data fabrication and one published by a scholar accused of sexual harassment. Note that, the order with which the two likelihood questions were presented was randomized.

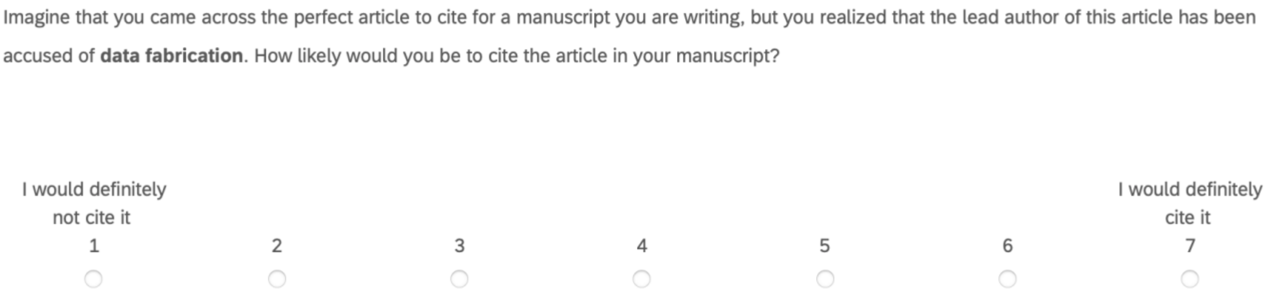

Fig. 9. Likelihood to cite an article published by a scholar accused of data fabrication question in the survey.

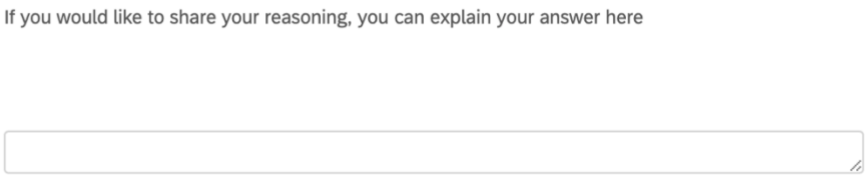

Fig. 10. Optional explanation of previous question.

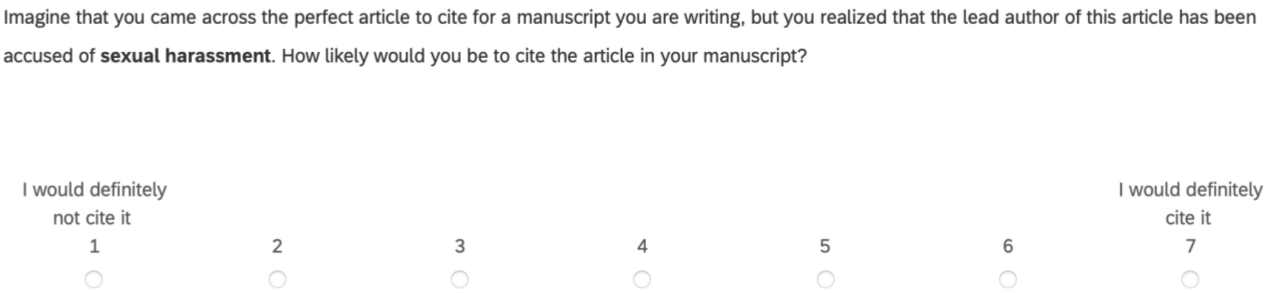

Fig. 11. Likelihood to cite an article published by a scholar accused of sexual harassment question in the survey.

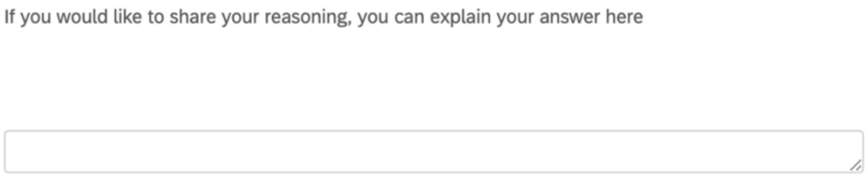

Fig. 12. Optional explanation of previous question.

Then, we asked participants to indicate which article, the one published by the scholar accused of data fabrication or the one published by the scholar accused of sexual harassment, they would be more likely to cite, if they were forced to cite one.

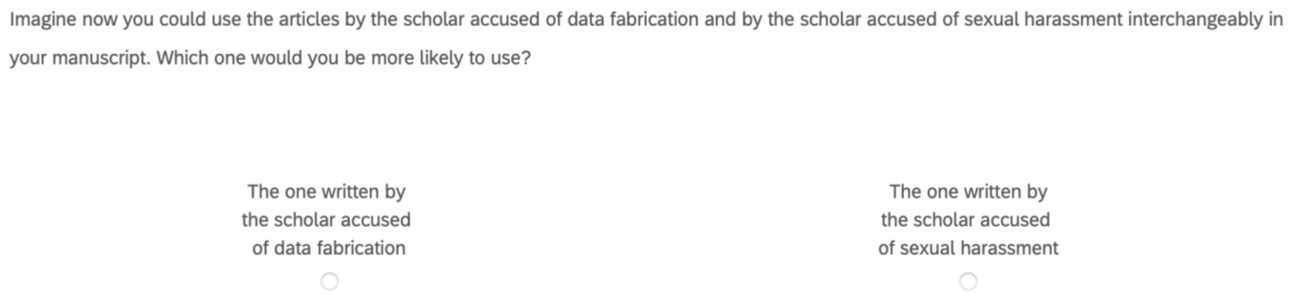
Imagine now you could use the articles by the scholar accused of data fabrication and by the scholar accused of sexual harassment interchangeably in your manuscript. Which one would you be more likely to use?

The one written by the scholar accused of data fabrication	The one written by the scholar accused of sexual harassment
○	○

Fig. 13. Which article they would be more likely to cite question in the survey.

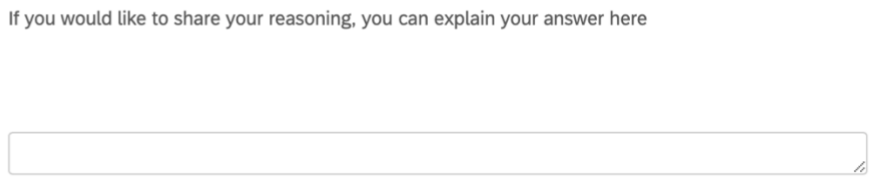
If you would like to share your reasoning, you can explain your answer here

Fig. 14. Optional explanation of previous question.

Results' Graphs

Likelihood to cite analysis on overall sample (N=240)

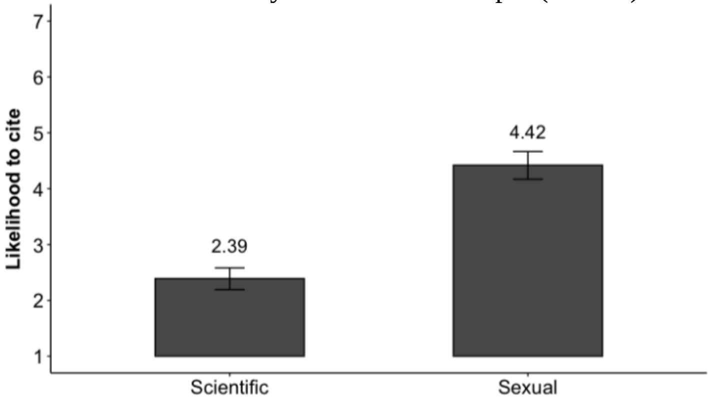

Fig. 15. Likelihood results on overall survey sample.

Analysis by Gender

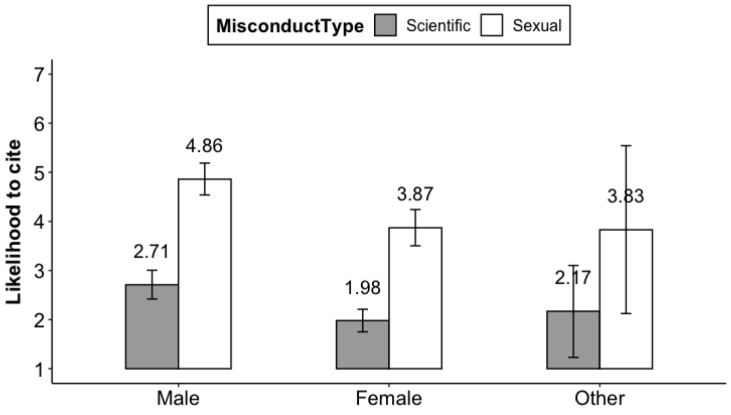

Fig. 16. Likelihood results by gender from the survey.

Analysis by Field

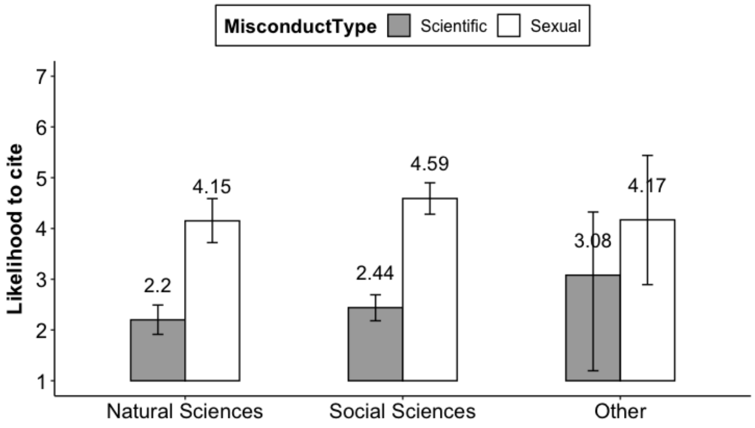

Fig. 17. Likelihood results by field from the survey.

Forced choice to cite analysis on overall sample (N=240)

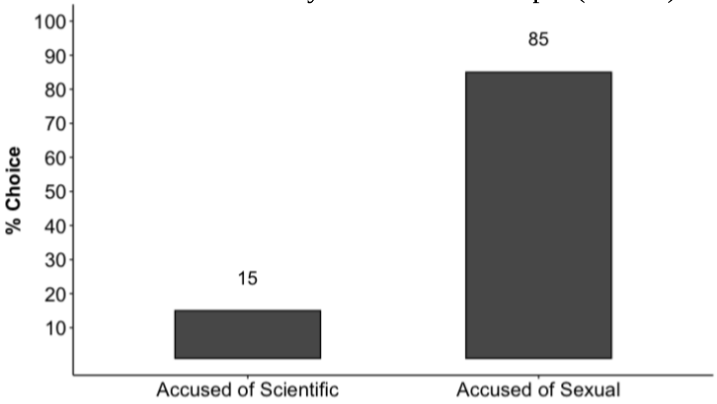

Fig. 18. Forced choice results on overall survey sample.

Analysis by Gender

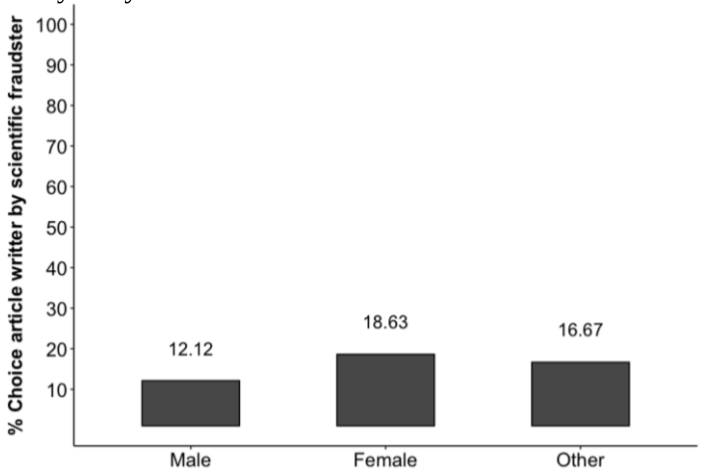

Fig. 19. Likelihood results by gender from the survey.

Analysis by Field

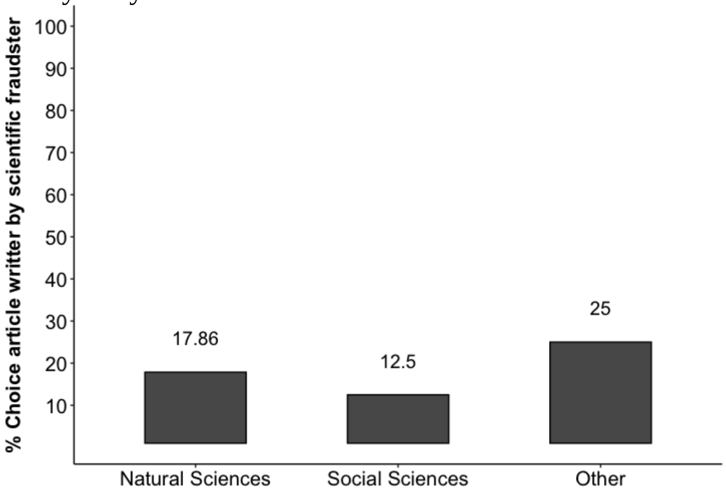

Fig. 20. Likelihood results by field from the survey.
